# GLP-1RA- and Incretin-Based Therapies Within Lifestyle Interventions for Adults with Overweight or Obesity: A Systematic Review and Meta-Analysis

**DOI:** 10.3390/nu18111781

**Published:** 2026-05-31

**Authors:** Alejandro Bruna-Mejias, Juan José Valenzuela-Fuenzalida, Gustavo Oyanedel, Julio Figueroa-Puig, Juan José Cabezas-Salgado, Mathias Orellana-Donoso, Gloria Cifuentes-Suazo, Juan Francisco Loro-Ferrer

**Affiliations:** 1 Programa de Doctorado en Investigación Aplicada a las Ciencias Sanitarias, Escuela de Doctorado, Universidad de Las Palmas de Gran Canaria (ULPGC), 35001 Las Palmas de Gran Canaria, Spain; 2 Escuela de Medicina, Facultad de Medicina, Universidad Andrés Bello, Viña del Mar 2520000, Chile; juan.kine.2015@gmail.com; 3 Department of Chemical and Biological Sciences, Faculty of Health Sciences, Universidad Bernardo O’Higgins, Santiago 8370993, Chile; 4 Faculty of Health Sciences, Universidad Autónoma de Chile, Santiago 8910060, Chile; g.oyanedelamaro@gmail.com; 5 Faculty of Health and Social Sciences, Universidad de Las Américas, Santiago 8370040, Chile; 6 School of Kinesiology, Faculty of Medicine, Universidad Mayor, Santiago 7500000, Chile; jifpuig@gmail.com; 7 Facultad de Medicina, Universidad Católica del Maule, Talca 3460000, Chile; jjcabezas@gmail.com; 8 School of Medicine, Universidad Finis Terrae, Santiago 7501015, Chile; mathor94@gmail.com; 9 Facultad de Medicina, Carrera de Odontología, Universidad Católica de la Santísima Concepción, Av. Alonso de Ribera 2850, Concepción 4090541, Chile; gbcifuentess@gmail.com; 10 Departamento Ciencias Clínicas, Universidad de Las Palmas de Gran Canaria (ULPGC), 35001 Las Palmas de Gran Canaria, Spain; juanfrancisco.loro@ulpgc.es

**Keywords:** GLP-1 receptor agonists, semaglutide, liraglutide, obesity, overweight, exercise, diet, lifestyle intervention, body composition, lean mass, fat mass, systematic review, meta-analysis

## Abstract

Background/Objectives: Glucagon-like peptide-1 receptor agonist (GLP-1RA)- and incretin-based therapies are now central to obesity management. Their clinical value, however, should be interpreted beyond total weight loss, because changes in fat mass, lean mass, physical function, and cardiometabolic risk may depend on the accompanying dietary, behavioral, and exercise co-interventions. This systematic review and meta-analysis evaluated GLP-1RA- and incretin-based therapies delivered within lifestyle interventions in adults with overweight or obesity. Methods: The protocol was registered in PROSPERO (CRD420261360837). PubMed/MEDLINE, Web of Science, Scopus, CINAHL, SPORTDiscus, and CENTRAL were searched from inception to the final search dates. Records were deduplicated in Zotero. Risk of bias was assessed using the Cochrane RoB 2 tool. Random-effects meta-analyses were estimated using restricted maximum likelihood with Hartung–Knapp adjustment when pooling was appropriate. Results: Across all database sources, 1651 records were identified. After removing 113 duplicate records and 212 records with an ineligible publication type before screening, 1326 records were screened. Seventy-seven reports were sought for retrieval, five were not retrieved, 72 were assessed at full text, and 48 reports corresponding to 35 independent parent trials or trial clusters were retained for qualitative synthesis. The primary kilogram-scale meta-analysis included eight independent comparisons and showed greater body-weight reduction with GLP-1RA/incretin-based therapy delivered within a lifestyle background than with placebo/control (mean difference [MD] −10.08 kg, 95% confidence interval [CI] −12.76 to −7.39; 95% prediction interval [PI] −17.86 to −2.29; *I*^2^ = 95.6%). Percentage body-weight change was analyzed separately across 11 independent comparisons and also favored GLP-1RA/incretin-based therapy (MD −9.53 percentage points, 95% CI −11.92 to −7.14; 95% PI −17.58 to −1.48; *I*^2^ = 95.4%). Conclusions: GLP-1RA- and incretin-based therapies delivered within lifestyle interventions are associated with clinically meaningful reductions in body weight in adults with overweight or obesity. Absolute and relative body-weight change metrics should remain analytically separate. The magnitude of benefit varies across trial contexts, and certainty remains limited by risk-of-bias concerns and considerable heterogeneity. Future trials should standardize the reporting of lifestyle co-interventions, body composition, adherence, physical-function outcomes, and safety monitoring.

## 1. Introduction

Obesity is a chronic, relapsing, and multifactorial disease with major implications for cardiometabolic health, physical function, quality of life, and health systems worldwide. According to the World Health Organization, in 2022 approximately 2.5 billion adults were living with overweight, including more than 890 million adults living with obesity. In the same year, 43% of adults worldwide were classified as overweight and 16% as having obesity [1,2]. These figures reflect the rapid global expansion of excess adiposity and reinforce the need for therapeutic strategies that are effective, sustainable, and capable of improving not only body weight but also metabolic health and functional outcomes.

In recent years, glucagon-like peptide-1 receptor agonist-based therapies have become a central pharmacological option for the management of obesity and related cardiometabolic disorders. Large randomized trials have shown that once-weekly semaglutide 2.4 mg, used as an adjunct to lifestyle intervention, produces substantial and sustained reductions in body weight in adults with overweight or obesity [3]. Similarly, tirzepatide, a dual glucose-dependent insulinotropic polypeptide and GLP-1 receptor agonist, has demonstrated marked weight re-duction in adults with obesity or overweight without diabetes [4]. These findings have shifted the therapeutic landscape of obesity from a model centered mainly on lifestyle advice toward an integrated chronic-care approach that combines pharmacotherapy with dietary, behavioral, and physical activity strategies.

Despite these advances, weight reduction alone is an incomplete marker of therapeutic success. Pharmacologically induced weight loss may include reductions in both fat mass and lean mass, and preservation of fat-free mass is clinically relevant because skeletal muscle contributes to resting energy expenditure, glucose disposal, mobility, strength, and long-term functional independence. Recent evidence suggests that GLP-1 receptor agonist-based therapies reduce total body weight and fat mass, but may also be associated with absolute reductions in lean mass, with lean mass accounting for approximately one quarter of total weight loss in some analyses [5]. This issue is particularly important when pharmacotherapy is used in populations at risk of sarcopenia, frailty, physical inactivity, or inadequate protein intake.

Lifestyle intervention remains a foundational component of obesity care. Dietary strategies are required to create an appropriate energy deficit and optimize nutrient quality, whereas exercise may contribute to weight maintenance, cardiometabolic improvement, physical fitness, and preservation of fat-free mass. Evidence from randomized trials suggests that combining pharmacotherapy with structured lifestyle components may provide additional benefits beyond either strategy alone. For example, semaglutide combined with intensive behavioral therapy and an initial low-calorie diet produced greater weight loss than placebo with the same behavioral intervention [6]. Likewise, exercise combined with liraglutide improved healthy weight-loss maintenance more than either intervention alone in adults with obesity after di-et-induced weight loss [7].

However, the clinical literature remains heterogeneous in the type, intensity, and re-porting of dietary and exercise co-interventions used alongside GLP-1 receptor agonist-based therapies. Some trials describe general lifestyle counseling, whereas others include structured dietary prescriptions, low-calorie diet phases, behavioral therapy, aerobic exercise, resistance training, or combined exercise programs. This variability makes it difficult to determine whether observed effects are attributable mainly to pharmacotherapy, to the lifestyle co-intervention, or to an interaction between both components. It also complicates the interpretation of outcomes beyond total body weight, particularly lean mass, fat mass, waist circumference, glycemic control, lipid profile, blood pressure, and other cardiometabolic endpoints.

Therefore, a systematic review focused on GLP-1 receptor agonist- and incretin-based therapies delivered within lifestyle interventions is warranted. This review aimed to clarify the extent to which these interventions affect body weight while also summarizing body composition and cardiometabolic outcomes in adults with overweight or obesity. This approach is clinically relevant because the long-term success of obesity treatment should be judged not only by the magnitude of weight loss, but also by the quality of that weight loss, preservation of lean tissue, metabolic improvement, and the potential sustainability of the intervention.

## 2. Methods

### 2.1. Study Design and Reporting Standards

This systematic review was designed to evaluate the effects of GLP-1 receptor agonist- and incretin-based therapies delivered within lifestyle interventions, including diet and/or exercise components, on body weight, body composition, and cardiometabolic outcomes in adults with overweight or obesity. The protocol was registered in PROSPERO (CRD420261360837). The review was reported in accordance with the Preferred Reporting Items for Systematic Reviews and Meta-Analyses (PRISMA) 2020 statement [8]. Methodological decisions regarding study selection, data extraction, risk-of-bias assessment, and synthesis followed current recommendations from the Cochrane Handbook for Systematic Reviews of Interventions where applicable [9].

### 2.2. Eligibility Criteria

Eligible studies were selected according to the population, intervention, comparator, outcomes, and study design framework. The population of interest comprised adults with overweight or obesity. The intervention of interest included GLP-1 receptor agonist-based pharmacological therapy, including GLP-1 receptor agonists and related incretin-based therapies, administered in combination with a dietary and/or exercise intervention. For the purposes of this review, GLP-1 receptor agonist-based therapies included GLP-1 receptor agonists and incretin-based or GLP-1 receptor-containing co-agonists, such as dual GIP/GLP-1 or GLP-1/glucagon receptor agonists, when GLP-1 receptor agonism was a core pharmacological mechanism. Dietary co-interventions included lifestyle counseling, caloric restriction, low-calorie diets, structured nutritional programs, and other diet-based strategies reported by the original studies. Exercise co-interventions included aerobic training, resistance training, combined exercise, structured physical activity, or exercise counseling, according to how each study defined the intervention.

Comparators included placebo, usual care, lifestyle intervention alone, pharmacotherapy alone, alternative pharmacological interventions, or other active control conditions, depending on the study design. Outcomes of interest included body weight, body mass index, waist circumference, fat mass, lean mass, fat-free mass, skeletal muscle mass, visceral adiposity, glycemic outcomes, lipid profile, blood pressure, and adverse events, when reported. Randomized clinical trials and controlled intervention studies were prioritized. Non-randomized designs were excluded from quantitative synthesis when they did not meet the eligibility standard for the review question.

### 2.3. Information Sources

A comprehensive electronic search was conducted from database inception to 30 April 2026 in PubMed/MEDLINE, Web of Science, Scopus, CINAHL, and SPORTDiscus. CENTRAL was searched via Ovid in EBM Reviews—Cochrane Central Register of Controlled Trials on 21 May 2026. Manual citation tracking was also used to identify potentially relevant studies not retrieved through electronic database searches.

Embase was not included because institutional access was unavailable. ClinicalTrials.gov and WHO ICTRP were removed from the final search plan before title and abstract screening, data extraction, risk-of-bias assessment, and synthesis. No date or language restrictions were applied to the CENTRAL search.

### 2.4. Search Strategy

The search strategy was developed using controlled vocabulary and free-text terms related to GLP-1 receptor agonist-based therapies, diet, exercise, lifestyle intervention, obesity, overweight, and relevant body-composition or cardiometabolic outcomes. Search syntax was adapted for each database according to its interface and field tags. The complete database-specific search strategies, including all final strings used for each source, are provided in Supplementary File S1.

The search was conducted in separate sets when required to preserve the conceptual structure of the strategy and improve retrieval control. The PubMed/MEDLINE, Web of Science, Scopus, CINAHL, and SPORTDiscus searches identified 361 records before deduplication. CENTRAL retrieved 1290 raw records; after applying the journal-article publication-type retention rule used for operational screening, 1078 CENTRAL records entered title and abstract screening. The complete database-specific strategies, including the CENTRAL/Ovid line-by-line strategy, are provided in Supplementary File S1.

### 2.5. Reference Management and Deduplication

All records retrieved from electronic database searches were exported and imported into Zotero for reference management. Duplicate records were identified using Zotero duplicate detection and manually verified when necessary. Across all database sources, 1651 records were identified. Before screening, 113 duplicate records and 212 records with an ineligible publication type were removed, leaving 1326 records for title and abstract screening. Reports selected for full-text assessment were crosswalked against the extraction matrix to identify independent parent trials, companion or secondary reports, overlapping analyses, exclusions, and reports not retrieved.

### 2.6. Study Selection

The deduplicated records were screened using a structured screening matrix. Title/abstract screening and full-text eligibility assessment were performed independently by two reviewers (JJVF and GO), and disagreements were resolved by a third reviewer (ABM). Each record was classified as Include, Exclude, or Maybe at the title and abstract stage. Records classified as Include or Maybe were moved to full-text retrieval. Reasons for exclusion were documented when sufficiently clear from title and abstract and were recorded in detail at the full-text stage.

For quantitative synthesis, eligible comparisons were retained only when extractable outcome data were available for the relevant scale and comparator. Outcomes reported on different scales were handled separately and were not statistically combined.

### 2.7. Data Extraction

Data were extracted independently by two reviewers (JJVF and GO) using a standardized form, with disagreements resolved by ABM. The extraction form captured study identification details, country, design, sample size, participant characteristics, GLP-1-based therapy type and dose, treatment duration, dietary and/or exercise co-intervention characteristics, comparator details, outcomes, time points, adverse events, funding source, and conflicts of interest, when available.

Because lifestyle co-interventions varied across trials, intervention and comparator characteristics were extracted separately from pharmacological exposure. Diet, exercise, behavioral counseling, supervision, and whether lifestyle support was balanced between trial arms were recorded to inform both quantitative synthesis and narrative interpretation. Trials with active structured lifestyle comparators, sequential exercise designs, metabolic ward feeding, or weight-loss-targeted treatment were not automatically excluded from the review; instead, their eligibility for meta-analysis was assessed at the outcome and comparison level.

### 2.8. Risk-of-Bias Assessment

Risk of bias was assessed independently by two reviewers (JJVF and GO) at the outcome level using the Cochrane Risk of Bias 2 tool for randomized trials [10], with disagreements resolved by ABM. The assessment focused on body-weight change, the primary outcome used for quantitative synthesis. Five domains were considered: bias arising from the randomization process, bias due to deviations from intended interventions, bias due to missing outcome data, bias in measurement of the outcome, and bias in selection of the reported result. Overall judgments were categorized as low risk of bias, some concerns, or high risk of bias. When multiple reports originated from the same parent trial, the risk-of-bias assessment was anchored to the parent trial and to the specific comparison/outcome used in the synthesis. Secondary or mechanistic reports were not used to inflate the number of independent studies.

### 2.9. Data Synthesis and Statistical Analysis

For continuous outcomes, treatment effects were summarized as mean differences (MDs) with 95% confidence intervals (CIs) when outcomes were reported on the same measurement scale. Body-weight change was defined as a primary continuous outcome and was analyzed preferentially on the kilogram scale. Studies reporting body-weight change in kilograms were pooled separately from studies reporting percentage body-weight change. Percentage body-weight change was therefore treated as a separate analysis and was not statistically combined with kilogram-based outcomes, avoiding inappropriate pooling of non-equivalent measurement scales.

Random-effects meta-analyses were performed using restricted maximum likelihood (REML) estimation with Hartung–Knapp adjustment for the confidence interval of the pooled effect. This approach was selected to provide a conservative estimate of uncertainty, particularly given the expected clinical and methodological heterogeneity across trials and the limited number of comparisons available for some outcomes. Statistical heterogeneity was quantified using *I*^2^, τ^2^, τ, Cochran Q, and the corresponding *p*-value. Prediction intervals were calculated to describe the range of effects that might be expected in a future comparable trial context and were interpreted cautiously.

All meta-analyses were conducted in RStudio (version 2026.04.0+526) using R and the metafor package. For body-weight outcomes, change-from-baseline values were preferred when available. Absolute kilogram change was prioritized for the primary meta-analysis, whereas percentage body-weight change was analyzed separately. When multiple follow-up time points were reported, the primary endpoint or the final time point of the intervention period was selected, according to the design and reporting of each parent trial. Data preparation involved selecting one eligible comparison per parent trial when required to avoid double counting, keeping kilogram-based and percentage-based outcomes analytically separate, and excluding comparisons from quantitative pooling when the required effect data were not extractable. No formal statistical imputation of missing outcome data was performed.

Eligibility for quantitative synthesis was determined at the comparison and outcome level. For the primary meta-analysis, only comparisons reporting body-weight change on the kilo-gram scale were pooled. Percentage body-weight change was analyzed separately and was not combined with kilogram-based outcomes. When multiple eligible arms or reports originated from the same parent trial, one clinically relevant comparison was selected to avoid double counting. Studies were retained for narrative synthesis when their design, comparator, fol-low-up duration, intervention structure, or outcome reporting precluded direct quantitative pooling. The rationale for inclusion in each quantitative synthesis is provided in Supplementary Table S1. Therefore, the pooled estimates should be interpreted primarily as the incremental effect of GLP-1 receptor agonist- or incretin-based therapy added to a lifestyle background, rather than as a formal estimate of pharmacotherapy-lifestyle synergy.

Meta-regression and formal subgroup analyses were not performed because the number of independent comparisons available for each pooled outcome was too small to support reliable moderator analyses. Potential sources of heterogeneity were therefore explored narratively, considering pharmacological agent, population context, diabetes or prediabetes status, com-parator structure, follow-up duration, treatment phase, and the intensity and supervision of lifestyle or exercise co-interventions.

### 2.10. Certainty of Evidence

The certainty of evidence was assessed using the GRADE approach for the main quantitative outcomes: body-weight change on the kilogram scale and percentage body-weight change. Because the quantitative evidence was derived from randomized trials, each outcome initially started at high certainty. Certainty was then rated down, when appropriate, across five domains: risk of bias, inconsistency, indirectness, imprecision, and publication bias. For each GRADE outcome, risk-of-bias judgments were based on the studies contributing data to that specific pooled estimate, rather than on all parent trials included in the systematic review. Risk-of-bias judgments were informed by the outcome-level RoB 2 assessment for body-weight change. Inconsistency was judged by considering the direction and magnitude of effects, overlap of confidence intervals, and clinical and methodological heterogeneity across trials. Indirectness was evaluated in relation to the review question, including population, pharmacological agent, comparator, lifestyle co-intervention, follow-up duration, and outcome scale. Imprecision was judged according to the width of the confidence interval, whether it crossed the line of no effect, and whether the interval remained compatible with clinically meaningful benefit. Publication bias was considered qualitatively because the number of comparisons was too small for reliable funnel-plot-based assessment. Body composition and cardiometabolic outcomes were not formally graded because differences in outcome definition, measurement method, intervention context, and reporting structure precluded identification of a single coherent estimate suitable for Summary of Findings presentation. The Summary of Findings table reports the final certainty rating and the main reasons for downgrading.

## 3. Results

### 3.1. Included Articles

**Figure 1:**
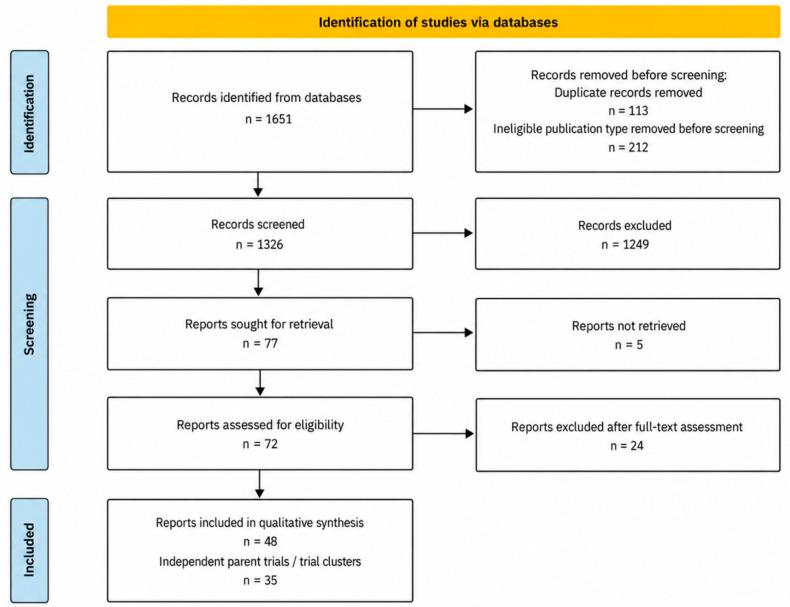
PRISMA 2020 flow diagram for study selection. Across all database sources, 1651 records were identified. After removal of 113 duplicate records and 212 records with ineligible publication type before screening, 1326 records were screened. Seventy-seven reports were sought for retrieval, five were not retrieved, 72 were assessed at full text, 24 were excluded with individualized reasons, and 48 reports corresponding to 35 independent parent trials or trial clusters were retained for qualitative synthesis.

Across all database sources, 1651 records were identified. Before title and abstract screening, 113 duplicate records and 212 records with an ineligible publication type were removed, leaving 1326 records for screening. Of these, 1249 records were excluded at title and abstract screening because they clearly did not meet the eligibility criteria. Seventy-seven reports were sought for retrieval, five reports were not retrieved, and 72 reports were assessed at full text. Twenty-four reports were excluded after full-text assessment with individualized PRISMA-compatible reasons, and 48 reports corresponding to 35 independent parent trials or trial clusters were retained for qualitative synthesis. The study selection process is summarized in Figure 1.

### 3.2. Characteristics of Included Studies

The included evidence base comprised 48 reports corresponding to 35 independent parent trials or trial clusters. Several reports were secondary, post hoc, mechanistic, pooled, or outcome-specific companion analyses of the same randomized parent trial; therefore, they were retained for qualitative or outcome-specific synthesis but were not counted as independent studies in the PRISMA flow diagram. Table 1 summarizes the parent-trial structure, population context, GLP-1RA- or incretin-based intervention, coparator/lifestyle framework, and outcome domains relevant to this review. Reports were handled at the parent-trial level to prevent double counting, particularly when they represented withdrawal designs, companion analyses, pooled analyses, or trial clusters already represented in the evidence base.

### 3.3. Intervention and Comparator Characteristics

Intervention and comparator characteristics are summarized in Table 2. Across the included trials, incretin-based pharmacotherapy was most often evaluated as an adjunct to lifestyle intervention, although the intensity, supervision, and balance of dietary and physical activity components varied substantially. Large phase 3 trials generally provided comparable lifestyle counseling to both intervention and placebo groups, allowing a cleaner estimation of the pharmacological effect added to lifestyle advice. By contrast, smaller mechanistic or disease-specific trials often used active lifestyle comparators, supervised exercise protocols, metabolic ward feeding, or weight-loss-targeted designs, limiting their suitability for direct quantitative pooling while still providing important mechanistic and contextual evidence.

### 3.4. Risk of Bias in Included Studies

Risk of bias was assessed at the outcome level for body-weight change using the Cochrane RoB 2 tool. The RoB 2 visualization was generated from the integrated body-weight evidence set, including parent trials or report clusters that contributed to the quantitative syntheses and those retained for narrative or contextual body-weight interpretation. GRADE judgments were applied separately to the specific studies contributing to each pooled estimate.

The main recurring concerns involved deviations from intended interventions, missing outcome data, and selection of the reported result. Measurement of body weight was consistently considered objective. A small number of active-comparator or mechanistic trials were judged at high overall risk of bias for body-weight effect estimation; these trials were retained for narrative or contextual interpretation when appropriate and were not allowed to distort the primary pooled estimates.

**Figure 2:**
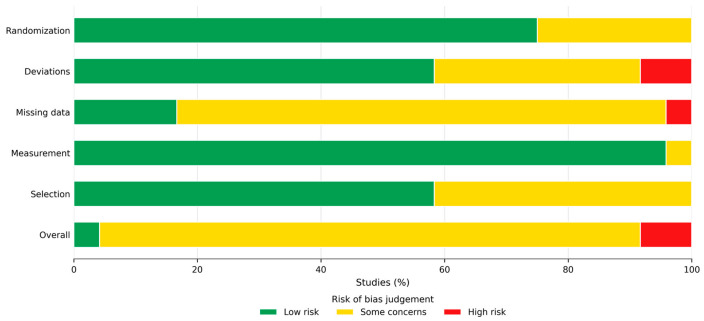
Risk-of-bias summary for body-weight change. The plot summarizes the proportion of included parent trials or report clusters with body-weight outcome data judged as low risk of bias, some concerns, or high risk of bias across RoB 2 domains and for the overall judgment. GRADE judgments were applied separately to the studies contributing to each pooled estimate.

The integrated RoB 2 summary is shown in Figure 2. The study-level traffic-light assessment is provided in Supplementary Figure S2 and Supplementary Table S2.

### 3.5. Body Weight Change, Kilogram Scale—Primary Analysis

Body-weight change reported in kilograms was considered the primary quantitative scale for this outcome. The final primary analysis was restricted to parallel placebo/control randomized trials with a balanced lifestyle background, extractable kilogram-scale effect estimates, and one independent comparison per parent trial. Designs estimating maintenance or randomized withdrawal effects, active lifestyle comparators, high-dose/dose-ranging contexts, disease-specific mechanistic trials, and companion reports were not combined with this primary induction model.

Eight independent comparisons contributed to the final kilogram-scale meta-analysis. Using a random-effects REML model with Hartung–Knapp adjustment, GLP-1 receptor agonist- or incretin-based therapy delivered within a lifestyle background produced greater body-weight reduction than placebo/control (MD −10.08 kg, 95% CI −12.76 to −7.39). The 95% prediction interval ranged from −17.86 to −2.29 kg. Negative mean differences indicate greater weight loss in the active intervention group. Between-study heterogeneity was considerable (*I*^2^ = 95.6%; τ^2^ = 9.55; Q(7) = 267.50, *p* < 0.001).

**Figure 3:**
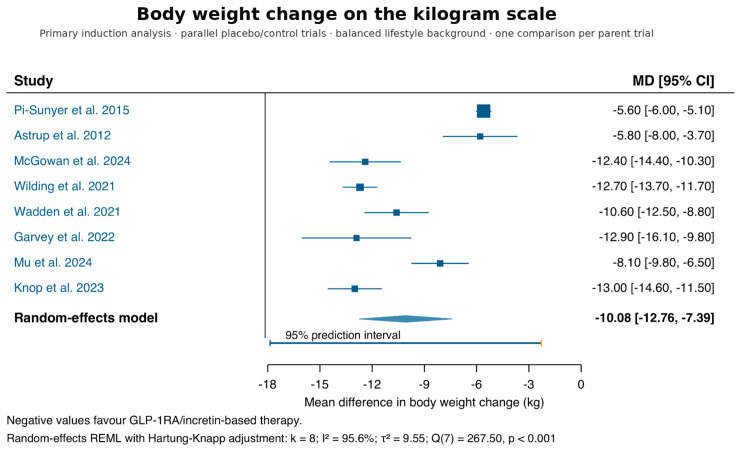
Forest plot for body-weight change on the kilogram scale. The primary induction analysis was restricted to parallel placebo/control trials with a balanced lifestyle background and one independent comparison per parent trial. Negative mean differences favour GLP-1 receptor agonist- or incretin-based therapy delivered within a lifestyle intervention. Squares represent study-level mean differences, horizontal lines represent 95% confidence intervals, the diamond represents the pooled random-effects estimate, the lower horizontal line represents the 95% prediction interval, and the vertical dashed line represents the line of no effect. The studies included in this analysis were Pi-Sunyer et al. (2015) [12], Astrup et al. (2012) [14], McGowan et al. (2024) [22], Wilding et al. (2021) [3], Wadden et al. (2021) [6], Garvey et al. (2022) [26], Mu et al. (2024) [28], and Knop et al. (2023) [34].

This finding supports a clinically meaningful average reduction in body weight when results are analyzed on the kilogram scale. However, the high heterogeneity indicates that the pooled estimate should not be interpreted as a single universal treatment effect across all GLP-1 receptor agonist- or incretin-based therapies, populations, doses, durations, and lifestyle contexts. Percentage body-weight change was not statistically combined with kilogram-based outcomes. The study-level estimates and pooled effect for the kilogram-scale primary analysis are shown in Figure 3.

### 3.6. Body Weight Change, Percentage Scale—Separate Analysis

Percentage body-weight change was analyzed separately because it represents a different measurement scale from absolute weight change in kilograms. The final percentage-scale analysis included 11 independent comparisons reporting percentage-point treatment effects and retained the same parent-trial independence rule used for the primary analysis.

Using a random-effects REML model with Hartung–Knapp adjustment, GLP-1 receptor agonist- or incretin-based therapy delivered within a lifestyle background was associated with greater percentage body-weight reduction than placebo/control (MD −9.53 percentage points, 95% CI −11.92 to −7.14). The 95% prediction interval ranged from −17.58 to −1.48 percentage points. Heterogeneity was considerable (*I*^2^ = 95.4%; τ^2^ = 11.91; Q(10) = 246.59, *p* < 0.001). The forest plot for this scale-specific analysis is provided in Supplementary Figure S1.

Therefore, the percentage-scale synthesis was directionally and statistically consistent with the kilogram-scale analysis while remaining analytically distinct. The result should be interpreted as a separate relative weight-change outcome rather than as a conversion or substitute for absolute kilogram change.

### 3.7. Certainty of Evidence and Summary of Findings

The certainty of evidence was assessed using the GRADE approach for the two body-weight outcomes included in the final quantitative synthesis. Each GRADE judgment was based only on the studies contributing data to the corresponding pooled estimate, not on all parent trials retained in the systematic review.

For kilogram-scale body-weight change, the certainty of evidence was rated as low. Although the pooled average effect was clinically meaningful and the confidence interval excluded the null, the body of evidence was downgraded for risk-of-bias concerns and serious inconsistency, mainly because heterogeneity remained considerable across pharmacological agents, trial contexts, populations, treatment durations, and lifestyle co-intervention structures.

For percentage body-weight change, the certainty of evidence was also rated as low. The pooled estimate favored the intervention and the confidence interval did not cross the null; however, the evidence was downgraded for risk-of-bias concerns and serious inconsistency because of substantial clinical and statistical heterogeneity. Publication bias was considered qualitatively because the number of comparisons remained insufficient for reliable funnel-plot-based assessment. The main GRADE judgments are summarized in Table 3, with the domain-level rationale provided in Supplementary Table S4.

No additional downgrade for imprecision was applied to either quantitative outcome because, in both final models, the 95% confidence interval and the 95% prediction interval remained on the beneficial side of the null. Publication bias was not formally testable because the number of comparisons remained below the threshold for reliable funnel-plot-based assessment.

### 3.8. Body Composition and Adiposity Outcomes

The availability of body-composition outcomes and the corresponding measurement method or assessment context, when reported or extractable, are summarized in Supplementary Table S3. Across studies, body-composition assessment relied on heterogeneous approaches, including DXA, BIA, MRI, CT, metabolic chamber protocols, or study-specific adiposity measures, which supported a narrative rather than quantitative synthesis.

Overall, studies reporting body composition generally suggested that GLP-1 receptor agonist-based interventions were associated with reductions in adiposity, although the magnitude and interpretation of fat mass and lean mass changes depended strongly on the co-intervention context. In the S-LiTE trial, structured exercise was particularly relevant because the parent trial assessed weight-loss maintenance after diet-induced weight loss using liraglutide, exercise, or the combination of both. This trial provided important evidence that exercise may modify the quality of weight loss and weight-loss maintenance, particularly by improving fitness and body composition rather than total body weight alone.

Several smaller or mechanistic studies provided additional information on adiposity compartments. Khoo et al. evaluated adults with obesity and non-alcoholic fatty liver disease and included liver fat-related outcomes, making the study particularly relevant for hepatic and visceral adiposity rather than for pooled weight-loss estimation. Liu et al. reported visceral fat area and body-fat percentage using multifrequency bioelectrical impedance analysis in adults with obesity receiving liraglutide plus metformin or metformin alone, with both groups receiving standardized lifestyle guidance. In that study, improvements in visceral fat area and body-fat percentage occurred alongside reductions in body weight and metabolic parameters, although the use of metformin in both arms and BIA-based assessment require cautious interpretation [21].

The metabolic ward study by Corbin et al. provided the most detailed mechanistic assessment of body composition, energy expenditure, and substrate oxidation. Under controlled caloric restriction, SAR425899 was associated with greater reductions in body weight, fat mass, and fat-free mass than placebo, but this was partly attributed to a greater achieved energy deficit than planned. The study also showed a smaller reduction in body-composition-adjusted sleeping metabolic rate and greater fat oxidation with SAR425899, supporting its value as mechanistic evidence rather than as a long-term clinical weight-loss efficacy trial [23].

Taken together, the body-composition evidence suggests possible favorable changes in adiposity with GLP-1 receptor agonist-based therapies, but the available data are insufficient to determine whether these interventions consistently preserve lean mass across clinical contexts. The role of structured exercise appears clinically important, particularly for improving physical fitness and potentially protecting fat-free mass during weight reduction. However, differences in measurement methods, including DXA, BIA, MRI, and metabolic chamber protocols, prevented formal pooling.

### 3.9. Reporting of Cardiometabolic and Mechanistic Outcomes

Cardiometabolic outcomes were frequently reported, but the specific endpoints varied across trials. Most large placebo-controlled trials included glycemic variables, lipid parameters, blood pressure, and waist circumference, whereas smaller mechanistic studies focused on insulin sensitivity, beta-cell function, liver fat, platelet activation, fat oxidation, or metabolic adaptation.

Glycemic outcomes generally improved in parallel with body-weight reduction. STEP 10 provided high-quality evidence in adults with obesity and prediabetes: semaglutide 2.4 mg plus diet and physical activity counseling produced greater body-weight reduction than placebo and a higher proportion of participants reverted to normoglycemia at week 52. In that trial, the primary endpoints were percentage change in body weight and reversion to normoglycemia, and the reported treatment effect favored semaglutide for both outcomes [22].

In studies enrolling participants with type 2 diabetes or impaired glucose regulation, GLP-1 receptor agonist-based interventions were associated with improvements in glycemic markers, although the clinical context differed substantially. SCALE Diabetes and Mensberg et al. contributed evidence in type 2 diabetes, whereas Ingersen et al. focused on beta-cell secretory function and aerobic training. These studies were not directly comparable to the non-diabetic obesity trials but were important for interpreting glycemic and exercise-related mechanisms.

Liu et al. provided mechanistic evidence linking weight loss, visceral adiposity, and beta-cell stress. In that trial, both liraglutide plus metformin and metformin alone were combined with standardized lifestyle intervention, including a 500 kcal/day energy-deficit diet and 150 min/week of moderate-intensity aerobic exercise. The reduction in proinsulin was more strongly attributed to weight loss and visceral fat reduction than to a direct GLP-1RA effect, suggesting that improvements in beta-cell-related biomarkers may be mediated substantially by the magnitude and distribution of weight loss [21].

The included mechanistic studies also highlighted pathways beyond traditional cardiometabolic endpoints. Corbin et al. reported that SAR425899 increased fat oxidation and ketogenesis and attenuated selective metabolic adaptation under controlled caloric restriction, although its short duration and dual GLP-1/glucagon mechanism make it clinically distinct from standard GLP-1RA obesity trials [23]. In SCALE IBT, adherence to dietary self-monitoring, physical activity, and medication use was evaluated as a behavioral determinant of weight loss, supporting the interpretation that pharmacotherapy and lifestyle adherence may interact in determining the magnitude of response [18].

Overall, cardiometabolic findings were directionally favorable, particularly for glycemic outcomes, waist circumference, blood pressure, and selected lipid parameters. However, heterogeneity in populations, pharmacological agents, comparator conditions, intervention intensity, and outcome reporting precluded a pooled cardiometabolic meta-analysis.

### 3.10. Adverse Events and Tolerability

Adverse events were reported inconsistently across the included studies, and the level of detail varied between large phase 3 trials, smaller exercise-based trials, and mechanistic studies. Safety outcomes were therefore summarized narratively.

Across the larger placebo-controlled trials, gastrointestinal adverse events were the most commonly reported tolerability issue, consistent with the known safety profile of GLP-1 receptor agonist-based pharmacotherapy. STEP 10 reported serious adverse events in 12 participants receiving semaglutide 2.4 mg and six receiving placebo, with adverse events leading to treatment discontinuation in eight participants in the semaglutide group and one participant in the placebo group. The authors reported no new safety signals, and the tolerability profile was considered consistent with the GLP-1 receptor agonist class [22].

Smaller studies generally provided less robust safety information because of limited sample sizes and shorter follow-up. The metabolic ward study of SAR425899 monitored adverse events under highly controlled inpatient conditions, but its 19-day duration limits inference about long-term tolerability. Similarly, exercise-based or mechanistic trials were valuable for understanding intervention feasibility and physiological effects, but they were not powered to detect uncommon adverse events.

Overall, the safety evidence did not suggest unexpected tolerability patterns beyond those already recognized for GLP-1 receptor agonist-based therapies. Nevertheless, differences in dose, treatment duration, comparator type, diabetes status, and intensity of lifestyle co-intervention limit direct comparison of adverse-event rates across studies. Future trials evaluating GLP-1 receptor agonist-based therapy combined with structured exercise should report adverse events, treatment discontinuation, exercise-related events, and adherence to both pharmacological and lifestyle components in a standardized manner.

## 4. Discussion

### 4.1. Principal Findings

This systematic review evaluated the effects of GLP-1 receptor agonist- and incretin-based therapies delivered within lifestyle interventions, including diet and/or exercise components, on body weight, body composition, and cardiometabolic outcomes in adults with overweight or obesity. The final integrated evidence base indicates that these therapies, when delivered within a lifestyle background, produce clinically meaningful average reductions in body weight compared with placebo/control conditions.

In the primary kilogram-scale meta-analysis, eight independent comparisons showed a pooled mean difference of −10.08 kg in favor of the active intervention, with both the 95% confidence interval and the 95% prediction interval favoring GLP-1 receptor agonist- or incretin-based therapy. The separate percentage-scale analysis, based on 11 independent comparisons, also favored the active intervention, with a pooled mean difference of −9.53 percentage points. These findings strengthen the direction of the quantitative evidence while preserving the methodological separation between absolute and relative weight-change scales.

At the same time, heterogeneity was considerable in both syntheses. The pooled estimates should therefore be interpreted as average effects across heterogeneous trial settings rather than as a single universal treatment effect applicable to every agent, dose, formulation, population, treatment duration, or lifestyle co-intervention context.

### 4.2. Interpretation of the Kilogram-Scale and Percentage-Scale Findings

The primary kilogram-scale result is clinically relevant because a mean difference of approximately 10 kg may translate into meaningful improvements in obesity-related risk factors when achieved and maintained in appropriate clinical contexts. The contributing studies were restricted to parallel placebo/control trial contrasts with a balanced lifestyle background and extractable absolute weight-change estimates. Importantly, the analysis used only one comparison per parent trial to avoid double counting, strengthening the internal coherence of the quantitative synthesis.

The percentage-scale analysis was intentionally treated as separate and supplementary. Although percentage weight loss is commonly used in obesity trials and regulatory contexts, combining it statistically with kilogram-based outcomes would have been inappropriate. In the final analysis, the percentage-scale estimate also favored the intervention and no longer crossed the null, but it should still be interpreted as a distinct outcome scale rather than as a re-expression of the kilogram-scale effect.

The high degree of statistical heterogeneity observed in both quantitative syntheses is clinically plausible. Included trials differed in pharmacological agent, formulation, dose, treatment duration, diabetes or prediabetes status, comparator structure, treatment phase, geographic and ethnic context, and intensity of lifestyle co-interventions. The results therefore support a robust average direction of benefit, while the precise magnitude of benefit remains context specific.

### 4.3. Influence of Diet, Exercise, and Behavioural Co-Interventions

One of the central findings of this review is that the lifestyle context surrounding GLP-1 receptor agonist-based therapy varied substantially across trials. In large phase 3 placebo-controlled studies, dietary advice and physical activity counseling were generally provided to both active and control groups, allowing a cleaner estimation of the pharmacological effect added to a comparable lifestyle background. In contrast, smaller trials often included active lifestyle comparators, supervised exercise protocols, metabolic ward feeding, or weight-loss-targeted designs. These differences are clinically important because they influence both the magnitude and interpretation of weight loss.

Exercise-based studies were particularly relevant for understanding whether pharmacotherapy and physical training may interact beyond total body-weight reduction. The S-LiTE trial is central in this regard because it directly evaluated liraglutide, exercise, and their combination after diet-induced weight loss. This design is highly informative for clinical practice because the goal of obesity treatment is not only to induce weight loss but also to maintain it while preserving or improving physical function. Similarly, studies combining GLP-1 receptor agonist therapy with supervised exercise in type 2 diabetes provided mechanistic evidence suggesting that exercise may contribute to improvements in cardiorespiratory fitness, glycemic control, and body composition, even when weight loss is not the primary endpoint.

However, the heterogeneity of lifestyle prescriptions also limited quantitative pooling. Some trials used general lifestyle counseling, whereas others used intensive behavioral therapy, low-calorie diet run-ins, structured aerobic or resistance training, or metabolic ward feeding. This variability reinforces the need to describe diet and exercise components separately from pharmacological exposure. It also suggests that future trials should report lifestyle adherence, exercise dose, dietary prescription, protein intake, resistance training exposure, and behavioral support using standardized frameworks.

### 4.4. Body Composition and Quality of Weight Loss

The body-composition findings were not sufficiently homogeneous for meta-analysis, but they are clinically important. Total body-weight reduction does not distinguish between fat mass, lean mass, water, or other compartments. Relying exclusively on total weight loss may therefore obscure whether an intervention improves metabolic health while preserving functionally important lean tissue.

The available evidence generally suggested reductions in adiposity with GLP-1 receptor agonist-based interventions. Studies reporting fat mass, visceral fat, liver fat, or body-fat percentage tended to show favorable changes, although the magnitude and comparability of these outcomes varied according to the assessment method. DXA, BIA, MRI, and metabolic chamber protocols were not interchangeable, and several outcomes were reported only in mechanistic studies or companion analyses.

Lean mass and fat-free mass require particularly cautious interpretation. Some GLP-1 receptor agonist-based interventions may reduce absolute lean mass as part of total weight loss. This does not necessarily imply functional harm, but it raises an important clinical question: how can pharmacologically induced weight loss be optimized to maximize fat loss while preserving muscle mass and physical function? Structured exercise, especially resistance training or combined aerobic-resistance training, may be a key modifier. However, the included evidence was not sufficient to determine whether exercise consistently attenuates lean mass loss during GLP-1 receptor agonist-based therapy. This remains a major research gap.

### 4.5. Cardiometabolic and Mechanistic Outcomes

Cardiometabolic outcomes were directionally favorable across several studies, particularly for glycemic markers, waist circumference, blood pressure, and selected lipid parameters. These changes are biologically plausible, given the magnitude of weight loss and the known metabolic effects of GLP-1 receptor agonist-based therapies. In participants with prediabetes, STEP 10 provided high-quality evidence that semaglutide 2.4 mg improved both body weight and glycemic status, including reversion to normoglycemia. In participants with type 2 diabetes, trials combining GLP-1 receptor agonist therapy with exercise or lifestyle intervention reported improvements in glycemic outcomes, although these studies differed from non-diabetic obesity trials in population and therapeutic context.

The mechanistic studies included in this review help explain why weight loss alone may not fully capture the therapeutic effects of GLP-1 receptor agonist-based interventions. Some studies assessed insulin sensitivity, beta-cell function, proinsulin processing, platelet activation, hepatic fat, substrate oxidation, and metabolic adaptation. For example, metabolic ward evidence with SAR425899 suggested effects on sleeping metabolic rate, fat oxidation, and ketogenesis under controlled caloric restriction. However, this study differed from standard GLP-1 receptor agonist obesity trials because it involved a dual GLP-1/glucagon receptor agonist, short follow-up, and highly controlled feeding. It should therefore be interpreted as mechanistic evidence rather than direct evidence of long-term clinical weight-loss efficacy.

Taken together, the cardiometabolic and mechanistic evidence suggests that GLP-1 receptor agonist-based therapies may improve multiple obesity-related pathways, but the current evidence base does not yet allow robust pooled estimates for most non-weight outcomes. Future research should prioritize harmonized reporting of glycemic, lipid, blood pressure, body-composition, functional, and safety endpoints.

### 4.6. Risk of Bias and Certainty of Interpretation

The RoB 2 assessment showed that the body-weight evidence base was not uniformly at low risk of bias. Body weight was objectively measured across trials, but several comparisons retained some concerns because of missing outcome data, treatment discontinuation, deviations from intended interventions, or selective-reporting considerations. A small number of active-comparator or mechanistic trials were judged at high overall risk of bias for body-weight effect estimation. These judgments support a cautious interpretation of the overall evidence while preserving the distinction between studies contributing to the pooled estimates and studies retained for narrative or contextual synthesis.

Several large randomized placebo-controlled trials had strong internal validity, including blinding, prespecified endpoints, and objective outcome measurement. In contrast, some smaller, mechanistic, active-comparator, dose-ranging, disease-specific, or maintenance/withdrawal contexts were retained for qualitative synthesis or separate interpretation rather than being blended into the primary induction forest plot. This approach reduced the risk of double counting and protected the main synthesis from clinically inappropriate pooling.

The formal GRADE assessment supports a cautious interpretation of the quantitative findings. The certainty of evidence was rated as low for both kilogram-scale and percentage-scale body-weight change. Although both pooled estimates and their prediction intervals favored GLP-1 receptor agonist- or incretin-based therapy, certainty was downgraded because most contributing comparisons had at least some risk-of-bias concerns and heterogeneity remained considerable. These judgments reinforce that the findings are clinically meaningful at the average-effect level, but that the precise magnitude of benefit should not be overgeneralized across all agents, populations, doses, and lifestyle contexts.

### 4.7. Strengths and Limitations

This review has several strengths. First, it specifically focused on GLP-1 receptor agonist- and incretin-based therapies in the context of diet, exercise, and lifestyle intervention, rather than evaluating pharmacotherapy in isolation. Second, the final search strategy included CENTRAL via Ovid, increasing retrieval breadth for randomized trial evidence. Third, the review distinguished between reports and independent parent trials, reducing the risk of double counting. Fourth, kilogram-based and percentage-based weight-change outcomes were analyzed separately, avoiding inappropriate pooling of non-equivalent measurement scales. Fifth, intervention and comparator characteristics were extracted in detail, allowing a more clinically meaningful interpretation of lifestyle co-intervention heterogeneity. Finally, risk of bias was assessed at the outcome level using RoB 2, with body-weight change as the target outcome for quantitative synthesis.

Several limitations should also be acknowledged. First, between-study heterogeneity remained considerable in both pooled analyses despite conservative comparison-level adjudication. Second, formal meta-regression or subgroup analysis was not performed because the number of independent comparisons within clinically coherent subgroups remained insufficient for reliable moderator analysis. Third, lifestyle co-interventions varied widely across trials, including general counseling, structured dietary prescriptions, low-calorie diet run-ins, intensive behavioral therapy, supervised exercise, and metabolic ward protocols. Fourth, body-composition outcomes were reported inconsistently and measured using different methods, limiting the ability to pool fat mass, lean mass, or visceral adiposity. Fifth, some studies were mechanistic, short-term, disease-specific, or used active lifestyle comparators, reducing direct comparability with large placebo-controlled obesity trials. Sixth, some included reports were secondary or companion analyses, requiring careful linkage to the parent trial to avoid inflating the number of independent studies. Seventh, Embase was not included because institutional access was unavailable, and trial registries were removed from the final search plan before screening, extraction, risk-of-bias assessment, and synthesis. These limitations should be considered when interpreting the precision and generalizability of the findings.

### 4.8. Clinical and Research Implications

Clinically, the findings support the use of GLP-1 receptor agonist-based therapies as part of an integrated obesity-management strategy that includes diet and physical activity. However, the review also highlights that lifestyle intervention should not be viewed as a passive background component. The type, intensity, supervision, and adherence to diet and exercise may influence not only the amount of weight lost but also the quality and sustainability of that weight loss.

For clinical practice, structured exercise may be especially important to preserve or improve physical function during pharmacologically induced weight reduction. Resistance training, aerobic training, and combined exercise may have different implications for lean mass, cardiorespiratory fitness, insulin sensitivity, and long-term weight maintenance. Future obesity trials evaluating GLP-1 receptor agonist-based therapies should therefore include standardized body-composition assessment, physical-function outcomes, dietary adherence, protein intake, exercise dose, and adverse events related to both medication and exercise.

From a research perspective, future studies should be designed to distinguish the independent and combined effects of pharmacotherapy, diet, and exercise. Trials should avoid underreporting lifestyle co-interventions, prespecify body-composition outcomes, and use harmonized time points and measurement scales. This would allow future meta-analyses to move beyond total body weight and better evaluate whether GLP-1 receptor agonist-based therapies produce high-quality, functionally favorable, and metabolically durable weight loss.

## 5. Conclusions

In this systematic review and meta-analysis, GLP-1 receptor agonist- and incretin-based therapy delivered within lifestyle interventions was associated with clinically meaningful reductions in body weight in adults with overweight or obesity. In the final primary kilogram-scale analysis, the pooled mean difference favored the intervention by approximately 10 kg. The separate percentage-scale analysis was consistent with this finding and favored the intervention by approximately 9.5 percentage points.

The certainty of evidence was rated as low for both kilogram-scale and percentage-scale body-weight change. The results therefore support a beneficial average effect on body weight, but the exact magnitude of benefit should be interpreted cautiously because of risk-of-bias concerns, considerable heterogeneity, variation in pharmacological agents and formulations, differences in lifestyle co-interventions, comparator type, follow-up duration, population context, and outcome reporting.

Beyond total body weight, the available narrative evidence suggests possible favorable changes in adiposity and selected cardiometabolic outcomes, particularly glycemic markers, waist circumference, blood pressure, and selected lipid parameters. However, the effects on lean mass, fat-free mass preservation, physical fitness, and regional adiposity remain insufficiently homogeneous for quantitative pooling.

Future trials should standardize dietary prescriptions, exercise dose, adherence reporting, body-composition methods, physical-function outcomes, adverse-event monitoring, and long-term follow-up to determine whether GLP-1 receptor agonist- and incretin-based pharmacotherapy combined with structured lifestyle support can improve not only the magnitude of weight loss but also its quality, durability, and cardiometabolic consequences.

## Figures and Tables

**Table 1:** Characteristics of included parent trials and report clusters.

Parent Trial/Report Cluster	Population/Clinical Context	GLP-1RA-Based Intervention	Comparator and Lifestyle Framework	Eligible Outcome Domains/Synthesis Role
Rubino et al. (2022) [11]	Adults with overweight or obesity without diabetes.	Semaglutide 2.4 mg once weekly compared with liraglutide 3.0 mg once daily.	Lifestyle counseling in the trial framework.	Body weight, waist circumference, and cardiometabolic markers. Classified as an active-comparator GLP-1RA trial.
Pi-Sunyer et al. (2015) [12]	Adults with overweight or obesity, including participants with prediabetes.	Liraglutide 3.0 mg plus lifestyle intervention.	Placebo plus the same reduced-calorie diet and increased physical activity framework.	Body weight, waist circumference, cardiometabolic outcomes, and early weight-loss response. Companion analyses were not counted as independent trials.
Lundgren et al. (2021) [7]	Adults with obesity after diet-induced weight loss.	Liraglutide, structured exercise, or the combination of liraglutide plus exercise.	Placebo/usual activity or exercise-alone arms according to the parent trial comparison.	Body weight, fat mass, lean mass, fitness, metabolic syndrome severity, glucose regulation, bone outcomes, appetite/physical activity behavior, and biomarkers. One comparison per parent trial was used in pooled analyses.
Davies et al. (2015) [13]	Adults with overweight or obesity and type 2 diabetes.	Liraglutide 3.0 mg or 1.8 mg plus diet and physical activity recommendation.	Placebo plus the same diet and physical activity recommendation.	Body weight, waist circumference, glycemic markers, and cardiometabolic outcomes. Not classified as a structured exercise-combination trial.
Astrup et al. (2012) [14]	Adults with obesity enrolled in a liraglutide weight-management trial/extension.	Once-daily liraglutide dose-ranging regimen with dietary and exercise counseling.	Control/comparator arms as reported in the parent trial.	Sustained weight loss, waist and cardiometabolic outcomes, and body composition at selected time points. Extension-phase data were handled cautiously.
Wadden et al. (2013) [15]	Adults with obesity after low-calorie-diet-induced weight loss.	Liraglutide 3.0 mg during weight-loss maintenance.	Placebo plus diet/exercise counseling throughout maintenance.	Body-weight maintenance, additional weight loss, waist circumference, and cardiometabolic risk factors. Independent parent trial.
Khoo et al. (2019) [16]	Adults with obesity and non-alcoholic fatty liver disease.	Liraglutide-induced weight-loss strategy.	Structured diet-and-exercise lifestyle modification.	Body weight, liver fat fraction, waist circumference, liver enzymes, cCK-18, and exercise maintenance. Analyzed as clinically distinct from placebo-controlled weight-loss trials.
Ingersen et al. (2023) [17]	Adults with type 2 diabetes and obesity.	Semaglutide administered concurrently with aerobic training.	Aerobic training without semaglutide.	Beta-cell secretory function, glucose tolerance, HbA1c, VO2max, and body composition. Outcome-specific extraction required.
Tronieri et al. (2020) [18]	Adults with overweight or obesity treated within an intensive behavioral therapy framework.	Liraglutide 3.0 mg plus intensive behavioral therapy.	Placebo plus intensive behavioral therapy.	Dietary self-monitoring, physical activity adherence, medication adherence, and weight-loss contribution. Used primarily for adherence/moderator synthesis.
Mensberg et al. (2017) [19]	Adults with type 2 diabetes, overweight or obesity.	Supervised exercise plus liraglutide.	Supervised exercise plus placebo.	HbA1c, body weight, body fat, VO2max, blood pressure, quality of life, and cardiac function. Companion report was not counted as an independent trial.
Simeone et al. (2018) [20]	Adults with obesity and prediabetes or early type 2 diabetes.	Liraglutide-induced weight-loss strategy.	Lifestyle-change-induced weight loss.	Platelet activation, lipid peroxidation, inflammation, insulin sensitivity, and adipose tissue distribution. Outcome-specific/mechanistic contribution.
Liu et al. (2025) [21]	Adults with obesity.	GLP-1RA plus metformin.	Metformin comparator, with standardized lifestyle guidance in both groups.	Body weight, visceral fat area, body fat percentage, lipids, glucose metabolism, and proinsulin processing. Metformin co-intervention was considered during synthesis.
McGowan et al. (2024) [22]	Adults with obesity and prediabetes.	Semaglutide 2.4 mg once weekly.	Placebo with diet and physical activity counseling.	Body weight, waist circumference, cardiometabolic markers, body composition subgroup, and safety. Final kg/% synthesis.
Corbin et al. (2023) [23]	Adults enrolled in a randomized metabolic ward study with calorie-reduced diet.	GLP-1/glucagon receptor agonism with calorie-reduced diet.	Placebo/control condition as reported in the metabolic ward trial.	Body weight, fat mass, fat-free mass, sleeping metabolic rate, 24 h energy expenditure, fat oxidation, and ketogenesis. Kept separate as a distinct pharmacological subclass.
Wilding et al. (2021) [3]	Adults with overweight or obesity without diabetes.	Semaglutide 2.4 mg once weekly.	Placebo plus lifestyle intervention with reduced-calorie diet and physical activity counseling.	Body weight, waist circumference, cardiometabolic markers, body composition subgroup, and safety. Final kg/% synthesis.
Davies et al. (2021) [24]	Adults with overweight or obesity and type 2 diabetes.	Semaglutide 2.4 mg or 1.0 mg once weekly.	Placebo plus lifestyle intervention.	Body weight, glycemic outcomes, waist circumference, cardiometabolic markers, and safety. Final % synthesis; T2D context.
Wadden et al. (2021) [6]	Adults with overweight or obesity without diabetes.	Semaglutide 2.4 mg once weekly.	Placebo plus intensive behavioral therapy and initial low-calorie diet.	Body weight, categorical weight loss, waist circumference, blood pressure, physical function, and safety. Final kg/% synthesis; IBT/LCD context.
Rubino et al. (2021) [25]	Adults with overweight or obesity without diabetes after semaglutide run-in.	Continued semaglutide 2.4 mg once weekly.	Switch to placebo; both groups continued lifestyle intervention.	Weight-loss maintenance and cardiometabolic outcomes. Randomized withdrawal design; analyze separately from baseline-randomized parallel trials.
Garvey et al. (2022) [26]	Adults with overweight or obesity without diabetes.	Semaglutide 2.4 mg once weekly.	Placebo plus behavioral intervention, reduced-calorie diet, and physical activity counseling.	Longer-term body weight and cardiometabolic outcomes. Final kg/% synthesis; long-duration context.
Kadowaki et al. (2022) [27]	East Asian adults with overweight or obesity, with or without type 2 diabetes.	Semaglutide 2.4 mg or 1.7 mg once weekly.	Placebo plus lifestyle recommendations.	Body weight, visceral fat, cardiometabolic outcomes, and safety.
Mu et al. (2024) [28]	Predominantly East Asian adults with overweight or obesity, with or without type 2 diabetes.	Semaglutide 2.4 mg once weekly.	Placebo plus diet and physical activity intervention.	Body weight, categorical weight loss, waist circumference, cardiometabolic outcomes, and safety. Final kg/% synthesis; population/duration context.
Bliddal et al. (2024) [29]	Adults with obesity and knee osteoarthritis.	Semaglutide 2.4 mg once weekly.	Placebo plus reduced-calorie diet and physical activity counseling.	Body weight, osteoarthritis-related outcomes, cardiometabolic markers, and safety. Condition-specific trial.
Lim et al. (2025) [30]	Asian adults with obesity without diabetes.	Semaglutide 2.4 mg once weekly.	Placebo plus reduced-calorie diet and increased physical activity.	Body weight, waist circumference, cardiometabolic markers, body composition subgroup, and safety. Final kg/% synthesis.
Kosiborod et al. (2023) [31]	Adults with obesity-related heart failure with preserved ejection fraction.	Semaglutide 2.4 mg once weekly.	Placebo; lifestyle framework as reported in the parent trial.	Body weight and clinical-function outcomes. Condition-specific parent trial; narrative or separate synthesis.
Wharton et al. (2025) [32]	Adults with obesity without diabetes.	Semaglutide 7.2 mg or 2.4 mg once weekly.	Placebo plus lifestyle intervention.	Body weight, waist circumference, cardiometabolic outcomes, MRI subset, and safety. High-dose trial; special context/sensitivity only.
Lingvay et al. (2025) [33]	Adults with obesity and type 2 diabetes.	Semaglutide 7.2 mg, 2.4 mg, or placebo.	Placebo plus lifestyle intervention.	Body weight, HbA1c, waist circumference, lipids, and safety. High-dose and T2D-specific context.
Knop et al. (2023) [34]	Adults with overweight or obesity without type 2 diabetes.	Oral semaglutide 50 mg once daily.	Placebo plus lifestyle intervention.	Body weight, categorical weight loss, cardiometabolic outcomes, and safety. Final kg/% synthesis; oral formulation context.
O’Neil et al. (2018) [35]	Adults with obesity without diabetes.	Dose-ranging daily semaglutide and liraglutide active comparator.	Placebo with dietary and physical activity counseling.	Body weight, waist circumference, cardiometabolic outcomes, and safety. Multi-arm dose-ranging trial; avoid double counting.
Zhao et al. (2024) [36]	Chinese adults with obesity or overweight and weight-related comorbidities, without diabetes.	Tirzepatide 10 mg or 15 mg once weekly.	Placebo plus lifestyle intervention.	Body weight, waist circumference, cardiometabolic outcomes, and safety.
Aronne et al. (2023) [37]	Adults with obesity or overweight after tirzepatide lead-in.	Continued tirzepatide.	Switch to placebo; both groups continued lifestyle intervention.	Weight-loss maintenance after tirzepatide lead-in. Randomized withdrawal design; analyze separately.
Garvey et al. (2025) [38]	Adults with overweight or obesity without diabetes.	Cagrilintide–semaglutide, semaglutide, or cagrilintide.	Placebo plus lifestyle intervention.	Body weight, waist circumference, body-composition subset, cardiometabolic outcomes, and safety.
Davies et al. (2025) [39]	Adults with overweight or obesity and type 2 diabetes.	Cagrilintide–semaglutide.	Placebo plus lifestyle intervention.	Body weight, glycemic outcomes, cardiometabolic markers, and safety.
Ariel et al. (2014) [40]	Adults with overweight/obesity and prediabetes.	Liraglutide with dietary energy restriction.	Placebo with matched dietary energy restriction.	Body weight, waist circumference, glycemic and lipoprotein outcomes; eligible parent trial.
Gudbergsen et al. (2021) [41]	Adults with overweight/obesity and knee osteoarthritis after diet-induced weight loss.	Liraglutide 3.0 mg daily.	Placebo in a weight-maintenance/lifestyle framework.	Body weight, knee osteoarthritis outcomes, anthropometry, and safety. Disease-specific maintenance context.
Moolla et al. (2025) [42]	Adults with MASLD without type 2 diabetes.	Liraglutide-induced weight loss.	Matched lifestyle-induced weight loss.	Liver fat, body composition, glucose homeostasis, lipids, de novo lipogenesis, and withdrawal effects. Mechanistic active-comparator evidence.

Abbreviations: BMI, body mass index; FPG, fasting plasma glucose; GLP-1RA, glucagon-like peptide-1 receptor agonist; HOMA-IR, homeostatic model assessment of insulin resistance; IBT, intensive behavioral therapy; NAFLD, non-alcoholic fatty liver disease; RCT, randomized controlled trial; T2D, type 2 diabetes; VO2max, maximal oxygen uptake.

**Table 2:** Intervention and comparator characteristics of included parent trials.

Parent Trial/Study	Pharmacological Intervention	Comparator	Dietary Component	Exercise/Physical Activity Component	Balance Between Groups	Interpretation for Synthesis
Rubino et al. (2022) [11]	Semaglutide 2.4 mg weekly or liraglutide 3.0 mg daily	Matched placebo groups/active comparator	Reduced-calorie diet, approximately 500 kcal/day deficit	Physical activity recommendation, commonly at least 150 min/week	Broadly balanced lifestyle background	Useful for pharmacological comparison under standardized lifestyle advice; active comparison was open-label.
Pi-Sunyer et al. (2015) [12]	Liraglutide 3.0 mg daily	Placebo	Reduced-calorie diet	Increased physical activity	Balanced	High-priority placebo-controlled trial for primary synthesis.
Lundgren et al. (2021) [7]	Liraglutide, exercise, or combined liraglutide plus exercise	Placebo/usual activity and/or exercise comparator	Low-calorie diet run-in followed by dietetic support	Structured moderate-to-vigorous exercise in exercise arms	Not fully balanced because exercise was randomized	Central trial for synergy question; one comparison per parent trial required.
Davies et al. (2015) [13]	Liraglutide 3.0 mg or 1.8 mg daily	Placebo	Approximately 500 kcal/day energy deficit	Physical activity recommendation, commonly 150 min/week	Balanced	Relevant for T2D subgroup; liraglutide 3.0 mg vs placebo is the clinically aligned contrast.
Astrup et al. (2012) [14]	Liraglutide dose-ranging regimen, including 3.0 mg	Placebo; open-label orlistat in original structure	Diet with approximately 500 kcal/day deficit	Exercise counseling	Balanced for liraglutide/placebo during controlled phase	Usable for kg synthesis at clean time point; extension data require caution.
Wadden et al. (2013) [15]	Liraglutide 3.0 mg after low-calorie diet-induced loss	Placebo	LCD run-in; reduced-calorie maintenance diet	Physical activity recommendation	Balanced after randomization	Strong trial for weight-loss maintenance; baseline should be randomization after LCD.
Khoo et al. (2019) [16]	Liraglutide 3.0 mg	Structured diet-exercise program	Structured restriction in lifestyle arm; general advice in liraglutide arm	Supervised/moderate physical activity target in lifestyle arm	Not balanced	Disease-specific mechanistic trial; not ideal for primary pooled placebo/control analysis.
Ingersen et al. (2023) [17]	Semaglutide followed by semaglutide plus aerobic training	Aerobic training alone	No primary structured dietary deficit	Supervised aerobic cycling, 3 sessions/week	Not fully balanced	Mechanistic evidence in T2D; not a clean parallel placebo-controlled obesity trial.
Tronieri et al. (2020) [18]	Liraglutide 3.0 mg plus intensive behavioral therapy	Placebo plus intensive behavioral therapy	1200–1800 kcal/day target with self-monitoring	Physical activity progressed from 100 to 250 min/week	Balanced	Relevant to behavioral intensity and weight-loss response within an intensive behavioral therapy framework.
Mensberg et al. (2017) [19]	Liraglutide 1.8 mg plus supervised exercise	Placebo plus supervised exercise	Participants instructed not to change diet	Three supervised 60 min sessions/week	Balanced for exercise exposure	Strong mechanistic trial for GLP-1RA plus exercise in T2D; weight is secondary.
Simeone et al. (2018) [20]	Liraglutide 1.8 mg	Lifestyle counseling	General advice in liraglutide arm; hypocaloric diet in lifestyle arm	Lifestyle arm targeted moderate activity ≥ 150 min/week	Not balanced	Mechanistic comparison of similar target weight loss achieved by different strategies.
Liu et al. (2025) [21]	Liraglutide plus metformin	Metformin alone	Standardized lifestyle guidance with 500 kcal/day deficit	150 min/week moderate aerobic exercise	Balanced lifestyle background; pharmacological contrast includes metformin	Useful for metabolic/proinsulin outcomes; comparator is not placebo.
McGowan et al. (2024) [22]	Semaglutide 2.4 mg weekly	Placebo	Individual diet counseling during treatment phase	Individual physical activity counseling	Balanced	High-priority phase 3 trial; provides kg and percentage weight outcomes.
Corbin et al. (2023) [23]	Dual GLP-1/glucagon receptor agonist SAR425899	Placebo	Metabolic ward diet with planned −1000 kcal/day deficit	Activity controlled in metabolic ward/calorimetry setting	Highly controlled, but achieved deficit differed	Mechanistic phase 1b evidence; not comparable with long-term obesity RCTs.
Wilding et al. (2021) [3]	Semaglutide 2.4 mg weekly	Placebo	Reduced-calorie diet, approximately 500 kcal/day deficit	Physical activity counseling, commonly 150 min/week	Balanced	Final kg/% synthesis; standard semaglutide context.
Davies et al. (2021) [24]	Semaglutide 2.4 mg or 1.0 mg weekly	Placebo	Lifestyle intervention	Physical activity counseling	Balanced	Final % synthesis; T2D context.
Wadden et al. (2021) [6]	Semaglutide 2.4 mg weekly	Placebo	Initial low-calorie diet plus intensive behavioral therapy	Progressive physical activity counseling	Balanced	Final kg/% synthesis; IBT/LCD context.
Rubino et al. (2021) [25]	Continued semaglutide 2.4 mg weekly	Switch to placebo	Reduced-calorie diet counseling	Physical activity counseling	Balanced after randomization	Randomized withdrawal design; separate maintenance/withdrawal synthesis or narrative.
Garvey et al. (2022) [26]	Semaglutide 2.4 mg weekly	Placebo	Reduced-calorie diet	Physical activity counseling	Balanced	Final kg/% synthesis; long-duration context.
Kadowaki et al. (2022) [27]	Semaglutide 2.4 mg or 1.7 mg weekly	Placebo	Lifestyle recommendations	Physical activity recommendations	Balanced	East Asian population; consider subgroup/contextual interpretation.
Mu et al. (2024) [28]	Semaglutide 2.4 mg weekly	Placebo	Diet intervention	Physical activity intervention	Balanced	Final kg/% synthesis; population/duration context.
Bliddal et al. (2024) [29]	Semaglutide 2.4 mg weekly	Placebo	Reduced-calorie diet	Physical activity counseling	Balanced	Knee osteoarthritis-specific population; narrative or stratified synthesis.
Lim et al. (2025) [30]	Semaglutide 2.4 mg weekly	Placebo	Reduced-calorie diet	Increased physical activity	Balanced	Final % synthesis; Asian/BMI-threshold context.
Kosiborod et al. (2023) [31]	Semaglutide 2.4 mg weekly	Placebo	As reported in parent trial	As reported in parent trial	Generally balanced	Obesity-related HFpEF; condition-specific clinical-function trial.
Wharton et al. (2025) [32]	Semaglutide 7.2 mg or 2.4 mg weekly	Placebo	Reduced-calorie diet	150 min/week physical activity	Balanced	High-dose semaglutide; special context/sensitivity only.
Lingvay et al. (2025) [33]	Semaglutide 7.2 mg or 2.4 mg weekly	Placebo	Lifestyle intervention	Lifestyle intervention	Balanced	High-dose and T2D-specific context.
Knop et al. (2023) [34]	Oral semaglutide 50 mg daily	Placebo	Lifestyle intervention	Lifestyle intervention	Balanced	Final kg/% synthesis; oral formulation context.
O’Neil et al. (2018) [35]	Daily semaglutide dose-ranging; liraglutide active comparator	Placebo and active comparator	Dietary counseling	Physical activity counseling	Balanced	Multi-arm phase 2 dose-ranging trial; one comparison rule required.
Zhao et al. (2024) [36]	Tirzepatide 10 mg or 15 mg weekly	Placebo	500 kcal/day deficit	At least 150 min/week physical activity	Balanced	Dual GIP/GLP-1 agonist; incretin-based evidence.
Aronne et al. (2023) [37]	Continued tirzepatide	Switch to placebo	Lifestyle intervention	Lifestyle intervention	Balanced after randomization	Randomized withdrawal design; analyze separately.
Garvey et al. (2025) [38]	Cagrilintide–semaglutide and component arms	Placebo	Lifestyle intervention	Lifestyle intervention	Balanced	Combination incretin/amylin pathway evidence; avoid dose-arm double counting.
Davies et al. (2025) [39]	Cagrilintide–semaglutide	Placebo	Lifestyle intervention	Lifestyle intervention	Balanced	T2D-specific combination therapy trial.
Ariel et al. (2014) [40]	Liraglutide	Placebo	Hypocaloric diet/energy restriction	Not central or as reported	Generally balanced for diet	Smaller cardiometabolic trial; narrative or scale-specific extraction.
Gudbergsen et al. (2021) [41]	Liraglutide 3.0 mg daily	Placebo	Post-diet weight-maintenance framework	Lifestyle context as reported	Balanced after randomization	Osteoarthritis-specific weight-maintenance trial; contextual synthesis.
Moolla et al. (2025) [42]	Liraglutide	Lifestyle-induced weight loss	Lifestyle arm used 500 kcal/day restriction	No specific exercise advice	Active comparator; not pharmacotherapy vs placebo	Mechanistic MASLD evidence; not primary pooled estimate.

**Table 3:** GRADE Summary of findings for body weight change outcomes.

Outcome	Summary of Findings and Certainty Judgment
Body-weight change, kg scale	8 comparisons; MD −10.08 kg (95% CI −12.76 to −7.39); 95% prediction interval −17.86 to −2.29 kg; *I*^2^ = 95.6%. Certainty: low. Downgraded for risk-of-bias concerns and serious inconsistency/heterogeneity; not downgraded for imprecision because both the confidence interval and prediction interval remained on the beneficial side of the null.
Body-weight change, percentage scale	11 comparisons; MD −9.53 percentage points (95% CI −11.92 to −7.14); 95% prediction interval −17.58 to −1.48 percentage points; *I*^2^ = 95.4%. Certainty: low. Downgraded for risk-of-bias concerns and serious inconsistency/heterogeneity; not downgraded for imprecision because both the confidence interval and prediction interval remained on the beneficial side of the null.

## Data Availability

The original contributions presented in this study are included in the article and Supplementary Materials. The analytic dataset and R code can be made available by the corresponding author upon reasonable request.

## Supplementary Materials

The following supporting information can be downloaded at: https://www.mdpi.com/article/10.3390/nu18111781/s1, Supplementary File S1: complete database-specific search strategies, including CENTRAL via Ovid, and supporting methodological materials; Figure S1: forest plot for body-weight change on the percentage scale; Table S1: eligibility of included studies for quantitative synthesis; Table S2: RoB 2 matrix for body-weight change; Table S3: outcome availability and measurement-method context for body-composition outcomes across included parent trials; Table S4: GRADE domain-level Summary of Findings for body-weight change; Figure S2: risk-of-bias traffic-light plot for body-weight change. The final quantitative synthesis used comparison-level adjudication and kept kilogram-scale and percentage-scale body-weight outcomes analytically separate.
