# Supplementary material for: GLP-1RA- and Incretin-Based Therapies Within Lifestyle Interventions for Adults with Overweight or Obesity: A Systematic Review and Meta-Analysis
Source: Nutrients. 2026 May 31;18(11):1781. doi: 10.3390/nu18111781 (PMC13259188; doi:10.3390/nu18111781)
Supplement: Supplementary file 1 [file nutrients-18-01781-s001.zip › nutrients-4342262-supplementary.pdf]

Supplementary File S1

Complete database-specific search strategies and supporting methodological materials

Effects of GLP-1 receptor agonist-based therapies within lifestyle interventions on body weight, body composition, and cardiometabolic outcomes in adults with overweight or obesity: a systematic review and meta-analysis

Search date: 30 April 2026.

The final search strategy included PubMed/MEDLINE, Web of Science Core Collection, Scopus, CINAHL, and SPORTDiscus. Search strings are reported below exactly as used/adapted for each database interface.

1) PubMed/MEDLINE (Advanced Search)

SET A — Core (sensitivo) + filtro RCT (sin fecha)

(("Obesity"[MeSH] OR obes*[tiab] OR overweight[tiab]) OR ("Diabetes Mellitus, Type 2"[MeSH] OR "type 2 diabet*"[tiab] OR T2DM[tiab])) AND ("Glucagon-Like Peptide 1 Receptor Agonists"[MeSH] OR semaglutide[tiab] OR liraglutide[tiab] OR tirzepatide[tiab] OR "glp-1 receptor agonist*"[tiab] OR incretin*[tiab] OR ozempic[tiab] OR wegovy[tiab] OR rybelsus[tiab] OR saxenda[tiab] OR victoza[tiab] OR mounjaro[tiab] OR zepbound[tiab]) AND ("Resistance Training"[MeSH] OR "Exercise Therapy"[MeSH] OR "Exercise"[MeSH] OR resistance train*[tiab] OR strength train*[tiab] OR "progressive resistance"[tiab] OR weight train*[tiab] OR aerobic train*[tiab] OR endurance train*[tiab] OR "aerobic exercise"[tiab] OR "moderate intensity"[tiab] OR "moderate-intensity"[tiab] OR "zone 2"[tiab] OR "zone-2"[tiab] OR "zona 2"[tiab] OR FATmax[tiab] OR "ventilatory threshold"[tiab] OR "lactate threshold"[tiab] OR VT1[tiab] OR LT1[tiab]) AND (randomized controlled trial[pt] OR controlled clinical trial[pt] OR randomi?ed[tiab] OR randomly[tiab] OR trial[ti]) NOT (animals[mh] NOT humans[mh])

SET B — Core + composición corporal (solo mapeo/priorización)

(("Obesity"[MeSH] OR obes*[tiab] OR overweight[tiab]) OR ("Diabetes Mellitus, Type 2"[MeSH] OR "type 2 diabet*"[tiab] OR T2DM[tiab])) AND ("Glucagon-Like Peptide 1 Receptor Agonists"[MeSH] OR semaglutide[tiab] OR liraglutide[tiab] OR tirzepatide[tiab] OR "glp-1 receptor agonist*"[tiab] OR incretin*[tiab] OR ozempic[tiab] OR wegovy[tiab] OR rybelsus[tiab] OR saxenda[tiab] OR victoza[tiab] OR mounjaro[tiab] OR zepbound[tiab]) AND ("Resistance Training"[MeSH] OR "Exercise Therapy"[MeSH] OR "Exercise"[MeSH] OR resistance train*[tiab] OR strength train*[tiab] OR "progressive resistance"[tiab] OR weight train*[tiab] OR aerobic train*[tiab] OR endurance train*[tiab] OR "aerobic exercise"[tiab] OR "moderate intensity"[tiab] OR "moderate-intensity"[tiab] OR "zone 2"[tiab] OR "zone-2"[tiab] OR "zona 2"[tiab] OR FATmax[tiab] OR "ventilatory threshold"[tiab] OR "lactate threshold"[tiab] OR VT1[tiab] OR LT1[tiab]) AND ("Body Composition"[MeSH] OR "Absorptiometry, Dual-Energy X-Ray"[MeSH] OR DXA[tiab] OR DEXA[tiab] OR "dual energy x-ray absorptiometry"[tiab] OR "bioelectrical impedance"[tiab] OR BIA[tiab] OR "fat-free mass"[tiab] OR "fat free mass"[tiab] OR "lean mass"[tiab] OR "lean body mass"[tiab]) AND (randomized controlled trial[pt] OR controlled clinical trial[pt] OR randomi?ed[tiab] OR randomly[tiab] OR trial[ti]) NOT (animals[mh] NOT humans[mh])

2.- Web of Science (Core Collection)

SET A — Core + RCT terms

TS=((obes* OR overweight OR "type 2 diabet*" OR T2DM OR DM2) AND (semaglutide OR liraglutide OR tirzepatide OR "GLP-1 receptor agonist*" OR incretin* OR ozempic OR wegovy OR rybelsus OR saxenda OR victoza OR mounjaro OR zepbound) AND (resistance train* OR strength train* OR weight train* OR "progressive resistance" OR aerobic train* OR "aerobic exercise" OR endurance train* OR "moderate intensity" OR "moderate-intensity" OR "zone 2" OR "zone-2" OR FATmax OR "ventilatory threshold" OR "lactate threshold" OR VT1 OR LT1) AND (random* OR trial OR placebo))

SET B — Core + composición corporal (mapeo)

TS=((obes* OR overweight OR "type 2 diabet*" OR T2DM OR DM2) AND (semaglutide OR liraglutide OR tirzepatide OR "GLP-1 receptor agonist*" OR incretin* OR ozempic OR wegovy OR rybelsus OR saxenda OR victoza OR mounjaro OR zepbound) AND (resistance train* OR strength train* OR weight train* OR "progressive resistance" OR aerobic train* OR "aerobic exercise" OR endurance train* OR "moderate intensity" OR "moderate-intensity" OR "zone 2" OR "zone-2" OR FATmax OR "ventilatory threshold" OR "lactate threshold" OR VT1 OR LT1) AND (DXA OR DEXA OR "dual energy x-ray absorptiometry" OR "bioelectrical impedance" OR BIA OR "fat-free mass" OR "fat free mass" OR "lean mass" OR "lean body mass") AND (random* OR trial OR placebo))

3.- Scopus

SET A — Core

TITLE-ABS-KEY((obes* OR overweight OR "type 2 diabet*" OR t2dm OR dm2) AND (semaglutide OR liraglutide OR tirzepatide OR "glp-1 receptor agonist*" OR incretin* OR ozempic OR wegovy OR rybelsus OR saxenda OR victoza OR mounjaro OR zepbound) AND (resistance train* OR strength train* OR weight train* OR "progressive resistance"OR aerobic train* OR "aerobic exercise" OR endurance train* OR "moderate intensity" OR "moderate-intensity" OR "zone 2" OR "zone-2" OR fatmax OR "ventilatory threshold" OR "lactate threshold" OR vt1 OR lt1) AND(random* OR trial OR placebo))

SET B — Core + composición corporal

TITLE-ABS-KEY((obes* OR overweight OR "type 2 diabet*" OR t2dm OR dm2) AND (semaglutide OR liraglutide OR tirzepatide OR "glp-1 receptor agonist*" OR incretin* OR ozempic OR wegovy OR rybelsus OR saxenda OR victoza OR mounjaro OR zepbound) AND (resistance train* OR strength train* OR weight train* OR "progressive resistance" OR aerobic train* OR "aerobic exercise" OR endurance train* OR "moderate intensity" OR "moderate-intensity" OR "zone 2" OR "zone-2" OR fatmax OR "ventilatory threshold" OR "lactate threshold" OR vt1 OR lt1) AND (DXA OR DEXA OR "dual energy x-ray absorptiometry" OR "bioelectrical impedance" OR BIA OR "fat-free mass" OR "fat free mass" OR "lean mass" OR "lean body mass") AND (random* OR trial OR placebo))

4.- CINAHL y SportDiscuss (EBSCOhost) — búsqueda avanzada

SET A — Core

((MH "Obesity+" OR TI obes* OR AB obes* OR TI overweight OR AB overweight) OR (MH "Diabetes Mellitus, Type 2+" OR TI "type 2 diabet*" OR AB "type 2 diabet*" OR TI T2DM OR AB T2DM)) AND ((MH "Glucagon-Like Peptide

1 Receptor Agonists+" OR TI semaglutide OR AB semaglutide OR TI liraglutide OR AB liraglutide OR TI tirzepatide OR AB tirzepatide OR TI "glp-1 receptor agonist*" OR AB "glp-1 receptor agonist*" OR TI incretin* OR AB incretin*)) AND ((MH "Resistance Training+" OR MH "Exercise Therapy+" OR MH "Exercise+") OR TI resistance N1 train* OR AB resistance N1 train* OR TI strength N1 train* OR AB strength N1 train* OR TI aerobic N1 train* OR AB aerobic N1 train* OR TI "zone 2" OR AB "zone 2" OR TI "zone-2" OR AB "zone-2" OR TI fatmax OR AB fatmax OR TI "ventilatory threshold" OR AB "ventilatory threshold" OR TI "lactate threshold" OR AB "lactate threshold") AND (PT clinical trial OR TI random* OR AB random* OR TI trial OR AB trial) NOT (MH "Animals+")

SET B — Core + composición corporal

(MH "Body Composition+" OR MH "Absorptiometry, Dual-Energy X-Ray+" OR TI DXA OR AB DXA OR TI DEXA OR AB DEXA OR TI "bioelectrical impedance" OR AB "bioelectrical impedance" OR TI BIA OR AB BIA OR TI "fat free mass" OR AB "fat-free mass" OR TI "lean mass" OR AB "lean mass" OR TI "lean body mass" OR AB "lean body mass")

Supplementary Figure S1. Forest plot for body weight change on the percentage scale

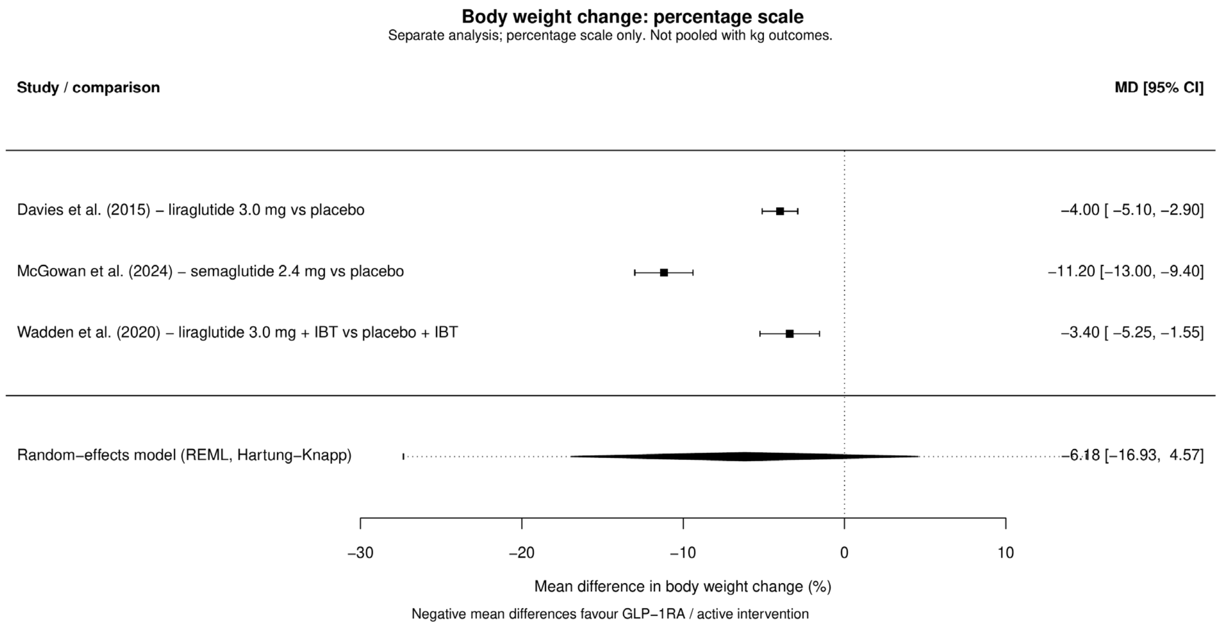

Figure S1. Forest plot for body weight change on the percentage scale. Percentage outcomes were analysed separately and were not pooled with kilogram-based outcomes. Negative mean differences favour GLP-1 receptor agonist-based therapy delivered within a lifestyle intervention. Squares represent study-level mean differences, horizontal lines represent 95% confidence intervals, the diamond represents the pooled random-effects estimate, the lower horizontal line represents the 95% prediction interval, and the vertical dashed line represents the line of no effect. The studies included in this analysis were Davies et al. (2015) [13], Wadden et al. (2020) [18], McGowan et al. (2024) [22], Wilding et al. (2021) [3], Davies et al. (2021) [24], Wadden et al. (2021) [6], Garvey et al. (2022) [26], Kadowaki et al. (2022) [27], Mu et al. (2024) [28], Knop et al. (2023) [34], and Lim et al. (2025) [30].

Supplementary Table S1. Eligibility of included studies for quantitative synthesis

Eligibility for quantitative synthesis was assessed at the comparison and outcome level. The primary quantitative synthesis was restricted to kilogram-based body weight change and used one comparison per parent trial to preserve statistical independence. Percentage body weight change was analysed separately and was not pooled with kilogram-based outcomes.

Parent trial / study	Unit available	Time point	Primary kg meta-analysis	Separate % analysis	Narrative only	Decision rationale
Pi-Sunyer et al. (2015) [12]	kg and %	Week 56	Yes	No	No	Main kg model; percentage outcome not counted separately to preserve the approved scale-specific mapping.
Astrup et al. (2012) [14]	kg	1 year	Yes	No	No	Main kg model; phase-II/dose-ranging context retained with sensitivity flag but approved for kg induction model.
McGowan et al. (2024) [22]	kg and %	Week 52	Yes	Yes	No	Approved for both kg and separate percentage models; analyses remain scale-specific.
Wilding et al. (2021) [3]	kg and %	Week 68	Yes	Yes	No	Phase 3 parent trial; balanced lifestyle background and extractable effect estimates.
Wadden et al. (2021) [6]	kg and %	Week 68	Yes	Yes	No	Intensive behavioural therapy/LCD context; approved with IBT flag.
Garvey et al. (2022) [26]	kg and %	Week 104	Yes	Yes	No	Long-duration phase 3 trial; approved with duration flag.
Mu et al. (2024) [28]	kg and %	Week 44	Yes	Yes	No	Predominantly East Asian population; approved with population/duration flag.
Knop et al. (2023) [34]	kg and %	Week 68	Yes	Yes	No	Oral semaglutide formulation; approved with formulation sensitivity flag.
Davies et al. (2015) [13]	%	Week 56	No	Yes	No	T2D-specific percentage outcome; analysed only in the separate percentage model.
Tronieri et al. (2020) [18]	%	Week 56	No	Yes	No / caution	IBT cluster; anchored to parent efficacy report and not counted as an independent companion report.
Davies et al. (2021) [24]	%	Week 68	No	Yes	No	T2D phase 3 evidence; percentage-scale model only.
Kadowaki et al. (2022) [27]	%	Week 68	No	Yes	No	East Asian phase 3 evidence; percentage-scale model only.
Lim et al. (2025) [30]	%	Week 44	No	Yes	No	Asian BMI-threshold trial; percentage-scale model only.
Lundgren et al. (2021) [7]; Wadden et al. (2013) [15]	kg/body composition	Maintenance context	No	No	Yes / supportive	Post-LCD or exercise-combination maintenance context; not blended with induction models.
Rubino et al. (2021) [25]; Aronne et al. (2023) [37]	kg and/or %	Withdrawal/maintenance	No	No	Yes / separate context	Randomized withdrawal after pharmacological run-in; estimates maintenance/withdrawal rather than induction.
Wharton et al. (2025) [32]; Lingvay et al. (2025) [33]	kg and %	High-dose context	No	No	Yes / sensitivity only	Semaglutide 7.2 mg not blended with standard-dose induction model.
Zhao et al. (2024) [36]; Garvey et al. (2025) [38]; Davies et al. (2025) [39]	% and related outcomes	Variable	No	No	Yes / subclass	Co-agonist/incretin-combination subclass; retained for qualitative interpretation, not GLP-1RA-only main model.

Parent trial / study	Unit available	Time point	Primary kg meta-analysis	Separate % analysis	Narrative only	Decision rationale
Bliddal et al. (2024) [29]; Kosiborod et al. (2023) [31]; Gudbergsen et al. (2021) [41]	kg and disease-specific outcomes	Variable	No	No	Yes	Disease-specific clinical contexts retained narratively to avoid distorting general obesity estimate.
Moolla et al. (2025) [42]; Khoo et al. (2019) [16]; Liu et al. (2025) [21]; Corbin et al. (2023) [23]; O'Neil et al. (2018) [35]	Variable	Variable	No	No	Yes	Mechanistic, active-comparator, dose-ranging, or non-comparable designs; not included in main pooled estimates.

Abbreviations: IBT, intensive behavioural therapy; LCD, low-calorie diet; NAFLD, non-alcoholic fatty liver disease; RoB 2, Cochrane Risk of Bias 2 tool; T2D, type 2 diabetes.

Supplementary Table S2. RoB 2 matrix for body weight change

Study	D1 Randomization	D2 Deviations	D3 Missing data	D4 Measurement	D5 Selection	Overall
Rubino et al. (2022) [11]	Low risk	Some concerns	Some concerns	Low risk	Some concerns	Some concerns
Pi-Sunyer et al. (2015) [12]	Low risk	Low risk	Some concerns	Low risk	Low risk	Some concerns
Lundgren et al. (2021) [7]	Low risk	Some concerns	Some concerns	Low risk	Low risk	Some concerns
Davies et al. (2015) [13]	Low risk	Low risk	Some concerns	Low risk	Low risk	Some concerns
Astrup et al. (2012) [14]	Low risk	Some concerns	Some concerns	Low risk	Some concerns	Some concerns
Wadden et al. (2013) [15]	Low risk	Low risk	Some concerns	Low risk	Low risk	Some concerns
Khoo et al. (2019) [16]	Some concerns	High risk	Some concerns	Low risk	Some concerns	High risk
Ingersen et al. (2023) [17]	Some concerns	Some concerns	Low risk	Low risk	Some concerns	Some concerns
Tronieri et al. (2020) [18]	Some concerns	Some concerns	Some concerns	Low risk	Some concerns	Some concerns
Mensberg et al. (2017) [19]	Some concerns	Low risk	Low risk	Low risk	Some concerns	Some concerns
Simeone et al. (2018) [20]	Some concerns	High risk	High risk	Low risk	Some concerns	High risk
Liu et al. (2025) [21]	Some concerns	Some concerns	Low risk	Some concerns	Some concerns	Some concerns
McGowan et al. (2024) [22]	Low risk	Low risk	Low risk	Low risk	Low risk	Low risk
Corbin et al. (2023) [23]	Low risk	Some concerns	Some concerns	Low risk	Some concerns	Some concerns
Wilding et al. (2021) [3]	Low risk	Low risk	Some concerns	Low risk	Low risk	Some concerns
Davies et al. (2021) [24]	Low risk	Low risk	Some concerns	Low risk	Low risk	Some concerns
Wadden et al. (2021) [6]	Low risk	Low risk	Some concerns	Low risk	Low risk	Some concerns
Rubino et al. (2021) [25]	Low risk	Low risk	Some concerns	Low risk	Low risk	Some concerns
Garvey et al. (2022) [26]	Low risk	Low risk	Some concerns	Low risk	Low risk	Some concerns
Kadowaki et al. (2022) [27]	Low risk	Low risk	Some concerns	Low risk	Low risk	Some concerns
Mu et al. (2024) [28]	Low risk	Low risk	Some concerns	Low risk	Low risk	Some concerns
Lim et al. (2025) [30]	Low risk	Low risk	Some concerns	Low risk	Low risk	Some concerns
Knop et al. (2023) [34]	Low risk	Low risk	Some concerns	Low risk	Low risk	Some concerns
O'Neil et al. (2018) [35]	Low risk	Some concerns	Some concerns	Low risk	Some concerns	Some concerns

D1: bias arising from the randomization process; D2: bias due to deviations from intended interventions; D3: bias due to missing outcome data; D4: bias in measurement of the outcome; D5: bias in selection of the reported result. The assessment was conducted at the outcome level for body weight change.

Supplementary Table S3. Outcome availability across included parent trials

Outcome availability was assessed at the parent-trial level. Companion reports were used to identify additional outcome domains but were not counted as independent trials. “Limited” indicates that the outcome was reported in a subgroup, secondary analysis, mechanistic report, or with insufficient homogeneity for quantitative pooling.

**Study**	**Body weight**	**BMI/waist**	**Fat mass/body fat**	**Lean mass/FFM**	**Visceral/liver adiposity**	**Glycemic outcomes**	**Lipids/BP**	**Fitness/exercise capacity**	**Adverse events**	**Main synthesis role**
Rubino et al. (2022) [11]	Yes	Yes	Limited/NR	NR	NR	Yes	Yes	NR	Yes	Narrative; potential sensitivity analysis
Pi-Sunyer et al. (2015) [12]	Yes	Yes	NR	NR	NR	Yes	Yes	NR	Yes	Primary kg synthesis
Lundgren et al. (2021) [7]	Yes	Yes	Yes	Yes	Yes/abdominal adiposity	Yes	Yes	Yes	Yes	Primary kg synthesis; key exercise-combination evidence
Davies et al. (2015) [13]	Yes	Yes	NR	NR	NR	Yes	Yes	NR	Yes	Separate percentage analysis
Astrup et al. (2012) [14]	Yes	Yes	Subgroup/limited	Subgroup/limited	NR	Yes	Yes	NR	Yes	Primary kg synthesis
Wadden et al. (2013) [15]	Yes	Yes	NR	NR	NR	Yes	Yes	NR	Yes	Primary kg synthesis
Khoo et al. (2019) [16]	Yes	Yes	Yes	NR/limited	Liver fat by MRI	Yes	Yes/liver enzymes	NR	Yes	Narrative; NAFLD-specific evidence
Ingersen et al. (2023) [17]	Yes	NR/limited	Yes	Yes	NR	Yes	Yes	Yes	Limited	Mechanistic/exercise-training evidence
Tronieri et al. (2020) [18]	Yes	NR/limited	NR	NR	NR	NR	NR	Physical activity adherence	NR/limited	Behavioural adherence and IBT synthesis

Mensberg et al. (2017) [19]	Yes	Yes	Yes	NR/limited	NR	Yes	Yes	Yes	Yes	GLP-1RA plus supervised exercise in T2D
Simeone et al. (2018) [20]	Yes	Yes	NR/limited	NR	SAT/VAT by imaging	Yes	Yes	NR	Limited	Mechanistic comparison of weight-loss strategies
Liu et al. (2025) [21]	Yes	Yes	Body fat percentage by BIA	NR/limited	Visceral fat area by BIA	Yes; proinsulin, HOMA-IR, QUICKI	Yes	NR	Yes	Metabolic/proinsulin mechanism
McGowan et al. (2024) [22]	Yes	Yes	NR	NR	NR	Yes; normoglycaemia, HbA1c, FPG	Yes	NR	Yes	Primary kg and separate percentage synthesis
Corbin et al. (2023) [23]	Yes	NR/limited	Yes	Yes/FFM	NR	Yes/metabolic biomarkers	Yes	Metabolic chamber activity control	Yes	Mechanistic metabolic ward evidence

Abbreviations: BIA, bioelectrical impedance analysis; BMI, body mass index; BP, blood pressure; FFM, fat-free mass; FPG, fasting plasma glucose; HOMA-IR, homeostatic model assessment of insulin resistance; IBT, intensive behavioural therapy; MRI, magnetic resonance imaging; NAFLD, non-alcoholic fatty liver disease; NR, not reported or not central to the report; QUICKI, quantitative insulin sensitivity check index; SAT, subcutaneous adipose tissue; T2D, type 2 diabetes; VAT, visceral adipose tissue.

Supplementary Table S4. GRADE domain-level Summary of Findings for body weight change

The certainty of evidence was assessed for the two body weight outcomes included in quantitative synthesis. Randomized evidence started at high certainty and was downgraded according to risk of bias, inconsistency, indirectness, imprecision, and publication bias. Each GRADE judgment was based only on the studies contributing data to the corresponding pooled estimate, not on all parent trials included in the systematic review.

**Outcome and contributing studies**	**Risk of bias**	**Inconsistency**	**Indirectness**	**Imprecision**	**Publication bias**	**Overall certainty**
Body weight change, kg scale Contributing comparisons: 8; MD -10.08 kg (95% CI -12.76 to -7.39); 95% PI -17.86 to -2.29; I² = 95.6%. Contributing studies: Pi-Sunyer et al. (2015) [12], Astrup et al. (2012) [14], McGowan et al. (2024) [22],	Serious: most contributing comparisons were judged as some concerns, mainly due to deviations from intended interventions or missing outcome data; body weight measurement itself was objective.	Serious: heterogeneity was considerable (I^2 = 92.8%; τ^2 = 7.55; Q(4) = 40.56, p < 0.001). The direction of the average effect favoured intervention, but the 95% prediction interval ranged from -15.70 to 1.18 kg and trials	Not serious to borderline: population and outcome matched the review question, but generalizability across all GLP-1RA-based therapies and structured exercise prescriptions remains limited.	Not serious for the pooled average effect: the 95% CI excluded the null and remained compatible with clinically meaningful benefit. The prediction interval crossed the null and was considered under inconsistency/heterogeneity rather than as a separate imprecision downgrade.	Undetected: not formally assessable because only five comparisons were available; absence of trial registries in the final search may increase residual uncertainty.	Low

Wilding et al. (2021) [3], Wadden et al. (2021) [6], Garvey et al. (2022) [26], Mu et al. (2024) [28], and Knop et al. (2023) [34].		differed in agent, dose context, lifestyle background, comparator structure, and treatment phase.				
Body weight change, percentage scale<br>Contributing comparisons: 11; MD -9.53 percentage points (95% CI -11.92 to -7.14); 95% PI -17.58 to -1.48; I^2 = 95.4%.<br>Contributing studies: Davies et al. (2015) [13], Wadden et al. (2020) [18], McGowan et al. (2024) [22], Wilding et al. (2021) [3], Davies et al. (2021) [24], Wadden et al. (2021) [6], Garvey et al. (2022) [26], Kadowaki et al. (2022) [27], Mu et al. (2024) [28], Knop et al. (2023) [34], and Lim et al. (2025) [30].	Serious: contributing evidence included some risk-of-bias concerns and relied on fewer comparisons.	Serious: heterogeneity was considerable (I^2 = 96.6%; τ^2 = 17.95; Q(2) = 50.59, $p < 0.001$). Heterogeneous trial contexts and reporting structures limited consistency of interpretation, and the 95% prediction interval ranged from -27.35% to 14.98%.	Serious to borderline: percentage change is clinically relevant but not interchangeable with kg change and was available in a restricted subset of trials.	Serious: the 95% CI was wide and crossed the line of no effect; the prediction interval was very wide, further supporting substantial uncertainty.	Undetected: not formally assessable because only three comparisons were available; absence of trial registries in the final search may increase residual uncertainty.	Very low

Abbreviations: CI, confidence interval; GLP-1RA, glucagon-like peptide-1 receptor agonist; GRADE, Grading of Recommendations Assessment, Development and Evaluation.

Supplementary Figure S2. Risk-of-bias traffic-light plot for body weight change

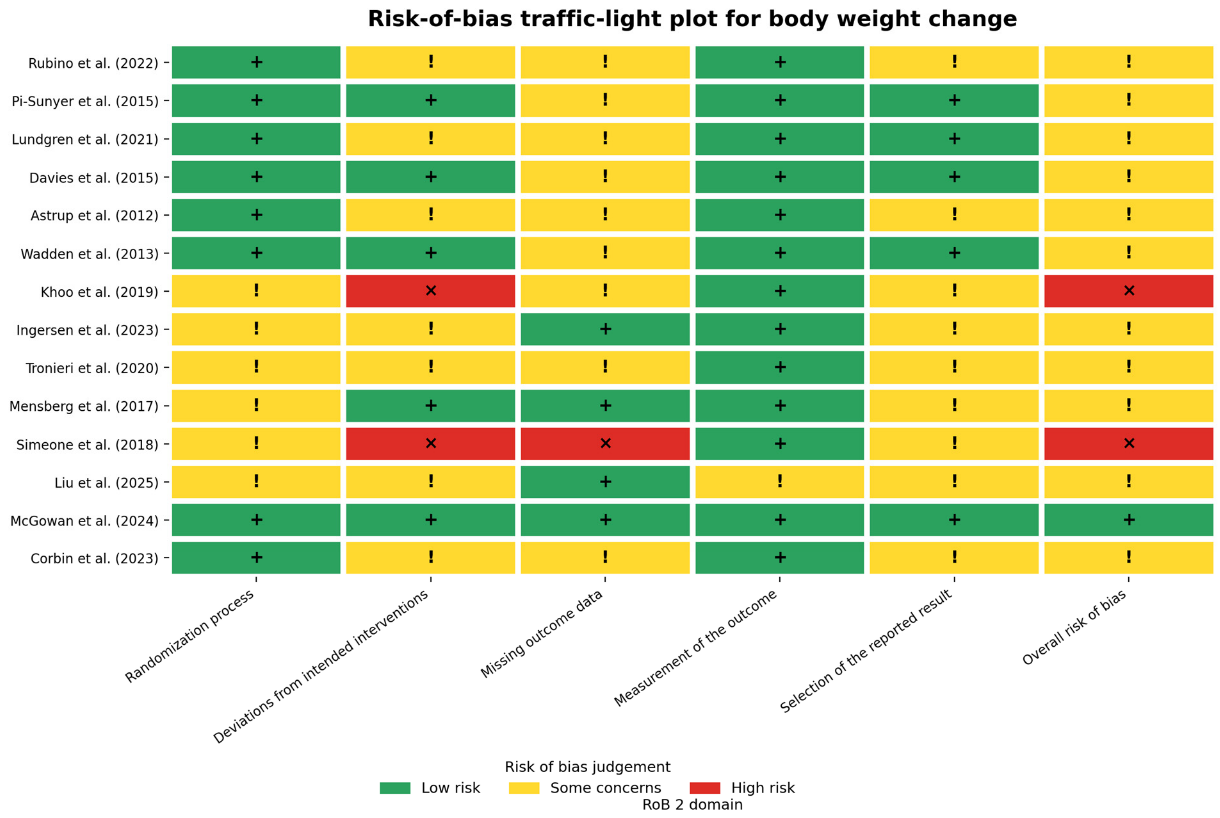

Supplementary Figure S2. Risk-of-bias traffic-light plot for body weight change using the Cochrane RoB 2 tool [10]. Each row represents one included parent trial or report cluster with body-weight outcome data, labelled by first author and publication year, and each column represents one RoB 2 domain plus the overall judgment. The traffic-light plot uses the integrated body-weight evidence set; studies retained for narrative/contextual synthesis are not treated as independent contributors to the pooled estimates. Symbols indicate low risk of bias (+), some concerns (!), and high risk of bias (x). The studies represented in the plot are cited in Supplementary Table S2 [3,6,7,11-30,34,35].

References

[3] Wilding, J.P.H.; Batterham, R.L.; Calanna, S.; Davies, M.; Van Gaal, L.F.; Lingvay, I.; McGowan, B.M.; Rosenstock, J.; Tran, M.T.D.; Wadden, T.A.; et al. Once-weekly semaglutide in adults with overweight or obesity. N. Engl. J. Med. 2021, 384, 989-1002. https://doi.org/10.1056/NEJMoa2032183.

[6] Wadden, T.A.; Bailey, T.S.; Billings, L.K.; Davies, M.; Frias, J.P.; Koroleva, A.; Lingvay, I.; O'Neil, P.M.; Rubino, D.M.; Skovgaard, D.; et al. Effect of subcutaneous semaglutide vs placebo as an adjunct to intensive behavioral therapy on body weight in adults with overweight or obesity: The STEP 3 randomized clinical trial. JAMA 2021, 325, 1403-1413. https://doi.org/10.1001/jama.2021.1831.

[7] Lundgren, J.R.; Janus, C.; Jensen, S.B.K.; Juhl, C.R.; Olsen, L.M.; Christensen, R.M.; Svane, M.S.; Bandholm, T.; Bojsen-Møller, K.N.; Blond, M.B.; et al. Healthy weight loss maintenance with exercise, liraglutide, or both combined. N. Engl. J. Med. 2021, 384, 1719-1730. https://doi.org/10.1056/NEJMoa2028198.

[10] Higgins, J.P.T.; Savović, J.; Page, M.J.; Elbers, R.G.; Sterne, J.A.C. Chapter 8: Assessing risk of bias in a randomized trial. In Cochrane Handbook for Systematic Reviews of Interventions, version 6.5; Higgins, J.P.T.; Thomas, J.; Chandler, J.; Cumpston, M.; Li, T.; Page, M.J.; Welch, V.A., Eds.; Cochrane: London, UK, 2024.

[11] Rubino, D.M.; Greenway, F.L.; Khalid, U.; O'Neil, P.M.; Rosenstock, J.; Sørrig, R.; Wadden, T.A.; Wizert, A.; Garvey, W.T.; for the STEP 8 Investigators. Effect of weekly subcutaneous semaglutide vs daily liraglutide on body weight in adults with overweight or obesity without diabetes: The STEP 8 randomized clinical trial. JAMA 2022, 327, 138-150. https://doi.org/10.1001/jama.2021.23619.

[12] Pi-Sunyer, X.; Astrup, A.; Fujioka, K.; Greenway, F.; Halpern, A.; Krempf, M.; Lau, D.C.W.; le Roux, C.W.; Violante Ortiz, R.; Jensen, C.B.; et al. A randomized, controlled trial of 3.0 mg of liraglutide in weight management. N. Engl. J. Med. 2015, 373, 11-22. https://doi.org/10.1056/NEJMoa1411892.

[13] Davies, M.J.; Bergenstal, R.; Bode, B.; Kushner, R.F.; Lewin, A.; Skjøth, T.V.; Andreasen, A.H.; Jensen, C.B.; DeFronzo, R.A.; for the NN8022-1922 Study Group. Efficacy of liraglutide for weight loss among patients with type 2 diabetes: The SCALE Diabetes randomized clinical trial. JAMA 2015, 314, 687-699. https://doi.org/10.1001/jama.2015.9676.

[14] Astrup, A.; Carraro, R.; Finer, N.; Harper, A.; Kunesova, M.; Lean, M.E.J.; Niskanen, L.; Rasmussen, M.F.; Rissanen, A.; Rössner, S.; et al. Safety, tolerability and sustained weight loss over 2 years with the once-daily human GLP-1 analogue, liraglutide. Int. J. Obes. 2012, 36, 843-854. https://doi.org/10.1038/ijo.2011.158.

[15] Wadden, T.A.; Hollander, P.; Klein, S.; Niswender, K.; Woo, V.; Hale, P.M.; Aronne, L.; on behalf of the NN8022-1923 Investigators. Weight maintenance and additional weight loss with liraglutide after low-calorie-diet-induced weight loss: The SCALE Maintenance randomized study. Int. J. Obes. 2013, 37, 1443-1451. https://doi.org/10.1038/ijo.2013.120.

[16] Khoo, J.; Hsiang, J.C.; Taneja, R.; Koo, S.H.; Soon, G.H.; Kam, C.J.; Law, N.M.; Ang, T.L. Randomized trial comparing effects of weight loss by liraglutide with lifestyle modification in non-alcoholic fatty liver disease. Liver Int. 2019, 39, 941-949. https://doi.org/10.1111/liv.14065.

[17] Ingersen, A.; Schmücker, M.; Alexandersen, C.; Graungaard, B.; Thorngreen, T.; Borch, J.; Holst, J.J.; Helge, J.W.; Dela, F. Effects of aerobic training and semaglutide treatment on pancreatic beta-cell secretory function in patients with type 2 diabetes. J. Clin. Endocrinol. Metab. 2023, 108, 2850-2863. https://doi.org/10.1210/clinem/dgad326.

[18] Wadden, T.A.; Tronieri, J.S.; Sugimoto, D.; Lund, M.T.; Auerbach, P.; Jensen, C.; Rubino, D. Liraglutide 3.0 mg and intensive behavioral therapy (IBT) for obesity in primary care: The SCALE IBT randomized controlled trial. Obesity 2020, 28, 529-536. https://doi.org/10.1002/oby.22726.

[19] Mensberg, P.; Nyby, S.; Jørgensen, P.G.; Storgaard, H.; Jensen, M.T.; Sivertsen, J.; Holst, J.J.; Kiens, B.; Richter, E.A.; Knop, F.K.; et al. Near-normalization of glycemic control with glucagon-like peptide-1 receptor agonist treatment combined with exercise in patients with type 2 diabetes. Diabetes Obes. Metab. 2017, 19, 172-180. https://doi.org/10.1111/dom.12797.

[20] Simeone, P.; Liani, R.; Tripaldi, R.; Di Castelnuovo, A.; Guagnano, M.T.; Tartaro, A.; Bonadonna, R.C.; Federico, V.; Cipollone, F.; Consoli, A.; et al. Thromboxane-dependent platelet activation in obese subjects with prediabetes or early type 2 diabetes: Effects of liraglutide- or lifestyle-change-induced weight loss. Nutrients 2018, 10, 1872. https://doi.org/10.3390/nu10121872.

[21] Liu, R.; Hou, D.; Leng, M.; Li, Z.; Zhang, Y.; Liu, L.; Wang, X.; Li, C. Weight loss mediates improvement in proinsulin processing during GLP-1 receptor agonist treatment. Diabetol. Metab. Syndr. 2025, 17, 286. https://doi.org/10.1186/s13098-025-01765-x.

[22] McGowan, B.M.; Bruun, J.M.; Capehorn, M.; Pedersen, S.D.; Pietiläinen, K.H.; Muniraju, H.A.K.; Quiroga, M.; Varbo, A.; Lau, D.C.W.; for the STEP 10 Study Group. Efficacy and safety of once-weekly semaglutide 2.4 mg versus placebo in people with obesity and prediabetes (STEP 10): A randomized, double-blind, placebo-controlled, multicentre phase 3 trial. Lancet Diabetes Endocrinol. 2024, 12, 631-642. https://doi.org/10.1016/S2213-8587(24)00182-7.

[23] Corbin, K.D.; Carnero, E.A.; Allerton, T.D.; Tillner, J.; Bock, C.P.; Luyet, P.P.; Göbel, B.; Hall, K.D.; Parsons, S.A.; Ravussin, E.; et al. GLP-1/glucagon receptor agonism associates with reduced metabolic adaptation and higher fat oxidation: A randomized trial. Obesity 2023, 31, 350-362. https://doi.org/10.1002/oby.23633.

[24] Davies, M.; Færch, L.; Jeppesen, O.K.; Pakseresht, A.; Pedersen, S.D.; Perreault, L.; Rosenstock, J.; Shimomura, I.; Viljoen, A.; Wadden, T.A.; et al. Semaglutide 2.4 mg once a week in adults with overweight or obesity, and type 2 diabetes (STEP 2): A randomized, double-blind, double-dummy, placebo-controlled, phase 3 trial. Lancet 2021, 397, 971-984. https://doi.org/10.1016/S0140-6736(21)00213-0.

[25] Rubino, D.; Abrahamsson, N.; Davies, M.; Hesse, D.; Greenway, F.L.; Jensen, C.; Lingvay, I.; Mosenzon, O.; Rosenstock, J.; Rubio, M.A.; et al. Effect of continued weekly subcutaneous semaglutide vs placebo on weight loss maintenance in adults with overweight or obesity: The STEP 4 randomized clinical trial. JAMA 2021, 325, 1414-1425. https://doi.org/10.1001/jama.2021.3224.

[26] Garvey, W.T.; Batterham, R.L.; Bhatta, M.; Buscemi, S.; Christensen, L.N.; Frias, J.P.; Jódar, E.; Kandler, K.; Rigas, G.; Wadden, T.A.; et al. Two-year effects of semaglutide in adults with overweight or obesity: The STEP 5 trial. Nat. Med. 2022, 28, 2083-2091. https://doi.org/10.1038/s41591-022-02026-4.

[27] Kadowaki, T.; Isendahl, J.; Khalid, U.; Lee, S.Y.; Nishida, T.; Ogawa, W.; Tobe, K.; Yamauchi, T.; Lim, S.; for the STEP 6 Investigators. Semaglutide once a week in adults with overweight or obesity, with or without type 2 diabetes, in an East Asian population (STEP 6): A randomized, double-blind, double-dummy, placebo-controlled, phase 3a trial. Lancet Diabetes Endocrinol. 2022, 10, 193-206. https://doi.org/10.1016/S2213-8587(22)00008-0.

[28] Mu, Y.; Bao, X.; Eliaschewitz, F.G.; Hansen, M.R.; Kim, B.T.; Koroleva, A.; Ma, R.C.W.; Yang, T.; Zu, N.; Liu, M.; for the STEP 7 Study Group. Efficacy and safety of once-weekly semaglutide 2.4 mg for weight management in a predominantly East Asian population with overweight or obesity (STEP 7): A double-blind, multicentre, randomized controlled trial. Lancet Diabetes Endocrinol. 2024, 12, 184-195. https://doi.org/10.1016/S2213-8587(23)00388-1.

[29] Bliddal, H.; Bays, H.; Czernichow, S.; Uddén Hemmingsson, J.; Hjelmesæth, J.; Hoffmann Morville, T.; Koroleva, A.; Skov Neergaard, J.; Vélez Sánchez, P.; Wharton, S.; et al. Once-weekly semaglutide in persons with obesity and knee osteoarthritis. N. Engl. J. Med. 2024, 391, 1573-1583. https://doi.org/10.1056/NEJMoa2403664.

[30] Lim, S.; Buranapin, S.; Bao, X.; Quiroga, M.; Park, K.H.; Kang, J.H.; Rinnov, A.R.; Suwanagool, A. Once-weekly semaglutide 2.4 mg in an Asian population with obesity, defined as BMI >=25 kg/m2, in South Korea and Thailand (STEP 11): A randomized, double-blind, placebo-controlled, phase 3 trial. Lancet Diabetes Endocrinol. 2025, 13, 838-847. https://doi.org/10.1016/S2213-8587(25)00164-0.

[31] Kosiborod, M.N.; Abildstrøm, S.Z.; Borlaug, B.A.; Butler, J.; Rasmussen, S.; Davies, M.; Hovingh, G.K.; Kitzman, D.W.; Lindegaard, M.L.; Møller, D.V.; et al. Semaglutide in patients with heart failure with preserved ejection fraction and obesity. N. Engl. J. Med. 2023, 389, 1069-1084. https://doi.org/10.1056/NEJMoa2306963.

[32] Wharton, S.; Freitas, P.; Hjelmesæth, J.; Kabisch, M.; Kandler, K.; Lingvay, I.; Quiroga, M.; Rosenstock, J.; Garvey, W.T.; on behalf of the STEP UP trial group. Once-weekly semaglutide 7.2 mg in adults with obesity (STEP UP): A randomized, controlled, phase 3b trial. Lancet Diabetes Endocrinol. 2025, 13, 949-963. https://doi.org/10.1016/S2213-8587(25)00226-8.

[33] Lingvay, I.; Bergenheim, S.J.; Buse, J.B.; Freitas, P.; Garvey, W.T.; Harder-Lauridsen, N.M.; Rosenstock, J.; Sahu, K.; Wharton, S.; on behalf of the STEP UP T2D trial group. Once-weekly semaglutide 7.2 mg in adults with obesity and type 2 diabetes (STEP UP T2D): A randomized, controlled, phase 3b trial. Lancet Diabetes Endocrinol. 2025, 13, 935-948. https://doi.org/10.1016/S2213-8587(25)00225-6.

[34] Knop, F.K.; Aroda, V.R.; do Vale, R.D.; Holst-Hansen, T.; Laursen, P.N.; Rosenstock, J.; Rubino, D.M.; Garvey, W.T.; for the OASIS 1 Investigators. Oral semaglutide 50 mg taken once per day in adults with overweight or obesity (OASIS 1): A randomized, double-blind, placebo-controlled, phase 3 trial. Lancet 2023, 402, 705-719. https://doi.org/10.1016/S0140-6736(23)01185-6.

[35] O'Neil, P.M.; Birkenfeld, A.L.; McGowan, B.; Mosenzon, O.; Pedersen, S.D.; Wharton, S.; Giwercman Carson, C.; Heerdegen Jepsen, C.; Kabisch, M.; Wilding, J.P.H. Efficacy and safety of semaglutide compared with liraglutide and placebo for weight loss in patients with obesity: A randomized, double-blind, placebo and active controlled, dose-ranging, phase 2 trial. Lancet 2018, 392, 637-649. https://doi.org/10.1016/S0140-6736(18)31773-2.

[36] Zhao, L.; Cheng, Z.; Lu, Y.; Liu, M.; Chen, H.; Zhang, M.; Wang, R.; Yuan, Y.; Li, X. Tirzepatide for weight reduction in Chinese adults with obesity: The SURMOUNT-CN randomized clinical trial. JAMA 2024, 332, 551-560. https://doi.org/10.1001/jama.2024.9217.

[37] Aronne, L.J.; Sattar, N.; Horn, D.B.; Bays, H.E.; Wharton, S.; Lin, W.Y.; Ahmad, N.N.; Zhang, S.; Liao, R.; Bunck, M.C.; et al. Continued treatment with tirzepatide for maintenance of weight reduction in adults with obesity: The SURMOUNT-4 randomized clinical trial. JAMA 2024, 331, 38-48. https://doi.org/10.1001/jama.2023.24945.

[38] Garvey, W.T.; Blüher, M.; Osorto Contreras, C.K.; Davies, M.J.; Winning Lehmann, E.; Pietiläinen, K.H.; Rubino, D.M.; Sbraccia, P.; Wadden, T.A.; Zeuthen, N.; et al. Coadministered cagrilintide and semaglutide in adults with overweight or obesity. N. Engl. J. Med. 2025, 393, 635-647. https://doi.org/10.1056/NEJMoa2502081.

[39] Davies, M.J.; Bajaj, H.S.; Broholm, C.; Eliasen, A.; Garvey, W.T.; le Roux, C.W.; Lingvay, I.; Lyndgaard, C.B.; Rosenstock, J.; Pedersen, S.D.; for the REDEFINE 2 Study Group. Cagrilintide-semaglutide in adults with overweight or obesity and type 2 diabetes. N. Engl. J. Med. 2025, 393, 648-659. https://doi.org/10.1056/NEJMoa2502082.
[41] Gudbergsen, H.; Overgaard, A.; Henriksen, M.; Wæhrens, E.E.; Bliddal, H.; Christensen, R.; Nielsen, S.M.; Boesen, M.; Knop, F.K.; Astrup, A.; et al. Liraglutide after diet-induced weight loss for pain and weight control in knee osteoarthritis: A randomized controlled trial. Am. J. Clin. Nutr. 2021, 113, 314-323. https://doi.org/10.1093/ajcn/nqaa328.
[42] Moolla, A.; Poolman, T.; Othonos, N.; Dong, J.; Smith, K.; Cornfield, T.; White, S.; Ray, D.W.; Mouchti, S.; Mózes, F.E.; et al. Randomised trial comparing weight loss through lifestyle modification with weight loss induced by glucagon-like peptide-1 receptor agonist therapy in people with MASLD without type 2 diabetes. JHEP Rep. 2025, 7, 101363. https://doi.org/10.1016/j.jhepr.2025.101363.
